# Defining genomic epidemiology thresholds for common-source bacterial outbreaks: a modelling study

**DOI:** 10.1016/S2666-5247(22)00380-9

**Published:** 2023-05

**Authors:** Audrey Duval, Lulla Opatowski, Sylvain Brisse

**Affiliations:** a Epidemiology and Modelling of Bacterial Escape to Antimicrobials Laboratory, Institut Pasteur, Université Paris Cité, Paris, France; b Anti-infective Evasion and Pharmacoepidemiology Team, CESP, Université Paris-Saclay, UVSQ, INSERM U1018, Montigny-le-Bretonneux, France; c Institut Pasteur, Université Paris Cité, Biodiversity and Epidemiology of Bacterial Pathogens, Paris, France

## Abstract

**Background:**

Epidemiological surveillance relies on microbial strain typing, which defines genomic relatedness among isolates to identify case clusters and their potential sources. Although predefined thresholds are often applied, known outbreak-specific features such as pathogen mutation rate and duration of source contamination are rarely considered. We aimed to develop a hypothesis-based model that estimates genetic distance thresholds and mutation rates for point-source single-strain food or environmental outbreaks.

**Methods:**

In this modelling study, we developed a forward model to simulate bacterial evolution at a specific mutation rate (μ) over a defined outbreak duration (*D*). From the distribution of genetic distances expected under the given outbreak parameters and sample isolation dates, we estimated a distance threshold beyond which isolates should not be considered as part of the outbreak. We embedded the model into a Markov Chain Monte Carlo inference framework to estimate the most probable mutation rate or time since source contamination, which are both often imprecisely documented. A simulation study validated the model over realistic durations and mutation rates. We then identified and analysed 16 published datasets of bacterial source-related outbreaks; datasets were included if they were from an identified foodborne outbreak and if whole-genome sequence data and collection dates for the described isolates were available.

**Findings:**

Analysis of simulated data validated the accuracy of our framework in both discriminating between outbreak and non-outbreak cases and estimating the parameters *D* and μ from outbreak data. Precision of estimation was much higher for high values of *D* and μ. Sensitivity of outbreak cases was always very high, and specificity in detecting non-outbreak cases was poor for low mutation rates. For 14 of the 16 outbreaks, the classification of isolates as being outbreak-related or sporadic is consistent with the original dataset. Four of these outbreaks included outliers, which were correctly classified as being beyond the threshold of exclusion estimated by our model, except for one isolate of outbreak 4. For two outbreaks, both foodborne *Listeria monocytogenes*, conclusions from our model were discordant with published results: in one outbreak two isolates were classified as outliers by our model and in another outbreak our algorithm separated food samples into one cluster and human samples into another, whereas the isolates were initially grouped together based on epidemiological and genetic evidence. Re-estimated values of the duration of outbreak or mutation rate were largely consistent with a priori defined values. However, in several cases the estimated values were higher and improved the fit with the observed genetic distance distribution, suggesting that early outbreak cases are sometimes missed.

**Interpretation:**

We propose here an evolutionary approach to the single-strain conundrum by estimating the genetic threshold and proposing the most probable cluster of cases for a given outbreak, as determined by its particular epidemiological and microbiological properties. This forward model, applicable to foodborne or environmental-source single point case clusters or outbreaks, is useful for epidemiological surveillance and may inform control measures.

**Funding:**

European Union Horizon 2020 Research and Innovation Programme.

## Introduction

Epidemics caused by exposure to a single common source (eg, unsafe food or contaminated water) are important targets of epidemiological surveillance and infection control strategies.^1^, ^2^ Rapid identification of the source enables outbreak control and is therefore crucial to public health. In the simplest and most common cases, a single pathogenic strain contaminates the source and subsequently causes infections (referred to as a clonal outbreak), which is often the case for contaminated food, water, or environmental sources. Such sources generally remain uncontaminated and often exist in the context of strong regulatory measures, especially in high-income countries; however, clonal outbreaks are still major causes of disease. Many countries have surveillance systems, to rapidly identify such outbreaks (eg*,* for foodborne pathogens such as *Salmonella* spp or *Listeria monocytogenes*), that use genome sequencing to identify related strains.^1^, ^2^ This strategy, named reverse epidemiology,^3^ forms the basis of surveillance systems used for foodborne pathogens such as PulseNet, one of the largest surveillance networks of bacterial genotypes worldwide.^1^ Molecular surveillance (ie, genetic fingerprinting) enables the detection of nearly identical infectious isolates and might trigger epidemiological investigations. These investigations include the search for case-associated risk factors such as consumption of a particular food item and microbiological analyses of suspected sources. Such investigations can lead to infection control measures that can prevent further cases.^1^, ^2^

**Research in context**

**Evidence before this study**

We searched PubMed for studies published in English from database inception to April 3, 2021, with the terms (threshold OR cut-off OR genetic relatedness) AND (outbreak) AND (cgMLST OR wgMLST OR SNPs) AND (microbial OR bacteria OR bacterial OR pathogen). We found 222 related articles. Most studies define a fixed single-nucleotide polymorphism (SNP) threshold that relates outbreak strains based on previous observations. One original study identifies outbreak clusters based on transmission events. However, this study relies on strong assumptions about molecular clock and transmission processes.

**Added value of this study**

Our study describes a new method based on a forward Wright-Fisher model to find the most appropriate genetic distance threshold to discriminate between outbreak and non-outbreak isolates. This method is fast and simple to use with only few assumptions, informed by outbreak duration and pathogen mutation rate. By using SNP or core genome multilocus sequence typing pairwise distances and sample collection dates of the outbreak of interest, the algorithm provides context-based guidance to separate outbreak strains from outliers.

**Implications of all the available evidence**

The fast and easy method developed in this study facilitates hypothesis-driven definitions of outbreak thresholds, in lieu of predefined thresholds. Defining clusters more accurately on the basis of an outbreak's specific epidemiological features, and estimating the most probable duration of the outbreak (time since initial source contamination), provides greatly needed precision for epidemiological surveillance and outbreak investigation. This novel approach might enable more efficient leveraging of molecular epidemiology data for the purposes of uncovering contamination sources.

Distinguishing case cluster isolates from sporadic ones has been the long-standing conundrum faced by molecular epidemiological surveillance. The identification of single-strain clusters of infections is confounded by a background of sporadic cases caused by exposure to unrelated sources. Defining a single strain typically uses a threshold of genetic distance, which discriminates between isolates that are related or unrelated to the same source or transmission event, and many attempts have been made to define such thresholds.^4^, ^5^ In the whole-genome sequencing era, thresholds have become smaller and more precise than with pre-genomic methods such as pulsed-field gel electrophoresis.^4^, ^6^, ^7^, ^8^, ^9^, ^10^ Threshold definition is usually based on the genetic variability observed within previous, well characterised outbreaks, an approach rooted in the epidemiological concordance principle.^2^, ^4^ However, consensus on the interpretation of molecular data for strain definition has not been reached.^4^, ^6^, ^11^

From an evolutionary perspective, bacteria that contaminate an initially sterile source can be considered as subpopulations of individual bacteria that have evolved from a single common ancestor (ie, the original strain) for a particular time period (ie, the duration since initial source contamination). Main factors that affect genetic distances between isolates include: (1) the amount of time between initial contamination of the source and the first infection, (2) the mutation rate of the pathogen's genomic markers, and (3) the sampling dates of infected patients. However, the genetic distance between outbreak isolates and the closest detected non-outbreak isolate depends on sampled genomes outside the contamination event. All these parameters considered, a threshold for one outbreak is unlikely to be applicable to another outbreak, even if they involve the same pathogen. For example, it is unrealistic to consider the same genetic threshold for an outbreak lasting 2 years versus 2 weeks, or for two pathogens with mutation rates that are orders of magnitude apart. Instead, using outbreak-specific thresholds defined based on the genetic diversity expected given their particular epidemiological contexts is likely to represent a more successful strategy. Attempts to ground threshold definition in evolutionary biology include the use of coalescent models,^7^ transmission models,^12^ and Bayesian most recent common ancestor models.^13^, ^14^

In this study, we aimed to develop a novel modelling framework, which we will refer to as sameStrain, to estimate genetic distance thresholds for single-strain outbreaks from a contaminated environmental or food source, based on outbreak-specific features (ie, pathogen genetic mutation rate and time since initial source contamination), by simulating the accumulation of mutations using these parameters. We embed this model into a Markov Chain Monte Carlo (MCMC) framework to estimate—from data including sampling dates and isolates' genetic variation—mutation rate or time since source contamination.

## Methods

### Definition of an outbreak

We define an outbreak (or cluster of cases) as a group of cases caused by a single strain (monoclonal outbreak), excluding co-occurring cases caused by genetically unrelated strains (ie, from other sources). Note that we focus here on environmental or food outbreaks with a single source (ie, excluding outbreaks involving human-to-human transmission).

### Identification of outbreak datasets

To identify datasets we searched PubMed and Google Scholar using the keywords “foodborne disease”, “foodborne investigation”, “foodborne illness”, “food source”, “data from outbreak investigation”, or “outbreak surveillance data”, published from database inception up until April 3, 2021, for studies published in English. We screened the articles for presence of genomic data, sampling dates, and clear epidemiological conclusions as to the inferred relationships of isolates with the infection cluster. We included articles that described only one pathogen.

### Evolutionary model

**Figure 1:**
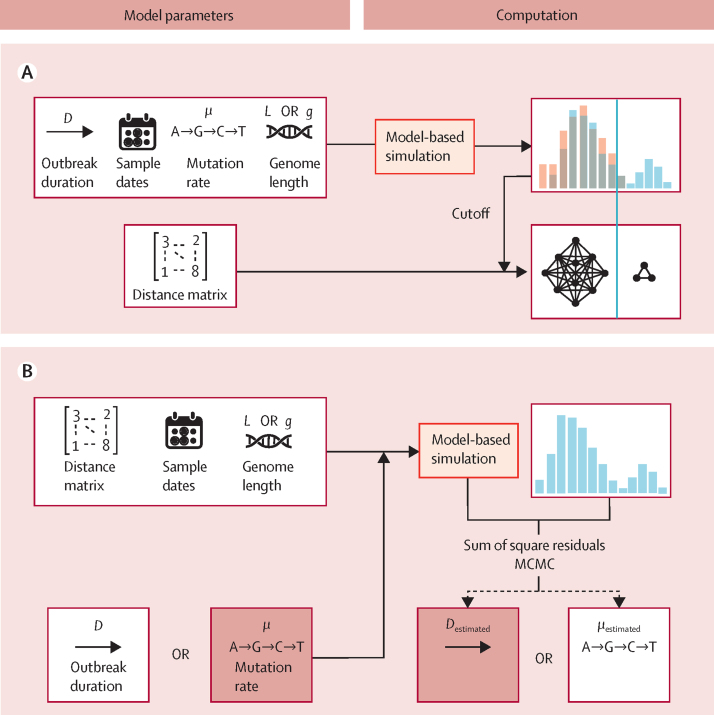
Description of the framework (A) Left, threshold computation inputs: genetic distance matrix *M*, duration of outbreak *D,* set of sample dates *S*_d_, number of substitutions per site per year μ, and sequence length *L* (if based on nucleotide sites), or number of genes *g* (if based on a gene-by-gene approach). Right, model-based simulation: the algorithm is initialised with a homogeneous population of individuals. At each time step, substitutions are drawn from a Poisson distribution, until *D* is reached. Samples are drawn randomly at the different observed sampling dates. A genetic threshold is defined using, for example, the 99th percentile of the distribution, and clusters of isolates are derived by single linkage clustering, leading to the ruling out of non-outbreak isolates (in this representation, the three isolates from the right would represent outliers unrelated to the cluster on the left, but would themselves form a cluster). (B) Left, the same model as in A is used to estimate *D* or μ using MCMC, based on the following inputs: the genetic distance matrix, the sampling dates, the sequence length, and either μ or *D* (depending on which one is estimated). MCMC=Markov Chain Monte Carlo.

Our evolutionary formalisation (figure 1A) is based on a Wright-Fisher forward model of haploid infectious agent evolution with constant population size.^15^, ^16^ Each simulation is initialised with a homogeneous population of an infectious agent characterised by five properties: (1) *L*, the genome length (in base pairs) or the average length of genes of multilocus sequence typing approaches; (2) *g*, the number of genes; (3) μ, the number of substitutions per site per year; (4) *D,* the duration (in days) of the outbreak, defined as the time elapsed between the initial contamination of the source, and the sampling date of the last isolate; and (5) *S*_d_, the set of isolate sampling dates, which is defined either directly from the source sampling dates or from infection sampling dates, ignoring incubation period and within-host evolution. Substitutions are introduced randomly at each time step, with their number following a Poisson distribution of parameter:

$$\lambda =\frac{\mu }{365}NLg$$

where *N* is the population size, in individual bacteria sampled uniformly with replacement from the population. After simulating over *D* days, we generated the final distribution of pairwise genetic distances by which one individual bacterium was randomly sampled from the observed sample set, *Sd*, on each sampling date. We then generated a distribution of pairwise genetic distances for these sampled individuals, which we used to define the genetic threshold value. Details of the sameStrain framework are provided in the appendix (p 2).

### Analysis of published outbreak datasets

We reviewed the published datasets of bacterial source-related outbreaks and analysed the datasets^7^ using our modelling framework. Inclusion criteria were: (1) an identified foodborne outbreak, (2) the availability of whole-genome sequence data, and (3) availability of collection dates for described isolates. In a first analysis, we extracted information on *D* from the original publications describing these outbreaks. We also used previously estimated values of μ and *g* for the corresponding pathogen from the literature. We label *D* and μ values taken from the literature as *D*_lit_ and μ_lit_, whereas those derived from our MCMC estimation (described later) are labelled as *D*_estimated_ and μ_estimated._

### Statistical analysis

To test the ability of the framework to distinguish between outbreak and non-outbreak cases, we ran a simulation study. We generated synthetic outbreaks from different combinations of *D* and μ (appendix p 6). We applied our framework to 171 independent simulated outbreaks generated with 19 distinct values of *D,* each combined with nine distinct values of μ and including simulated non-outbreak or sporadic isolates (appendix p 6). For each simulated outbreak, we assessed the global sensitivity and specificity of the framework (appendix p 3).

To address uncertainty underlying key model parameters, including the time since initial source contamination and the genetic mutation rate, our model was embedded into a Bayesian statistical inference framework to enable estimation of either the duration (*D*) or the substitution rate (μ) of studied outbreaks when unknown (figure 1B; appendix pp 2–4). Briefly, we estimate *D* or *μ* from the observed pairwise genetic distance matrix by using an MCMC algorithm. The simulated outbreaks described earlier were used to assess the ability of the model to estimate *D* and μ, and their impact on estimation of the genetic threshold. We used the 95% highest posterior density (95% HPD) intervals to assess accuracy of the estimates. We used the Kolmogorov-Smirnoff test statistic to compare real distributions with simulated distributions as a goodness of fit indicator. No ethical approval was needed for this study.

### Role of the funding source

The funder of the study had no role in study design, data collection, data analysis, data interpretation or writing of the report.

## Results

**Figure 2:**
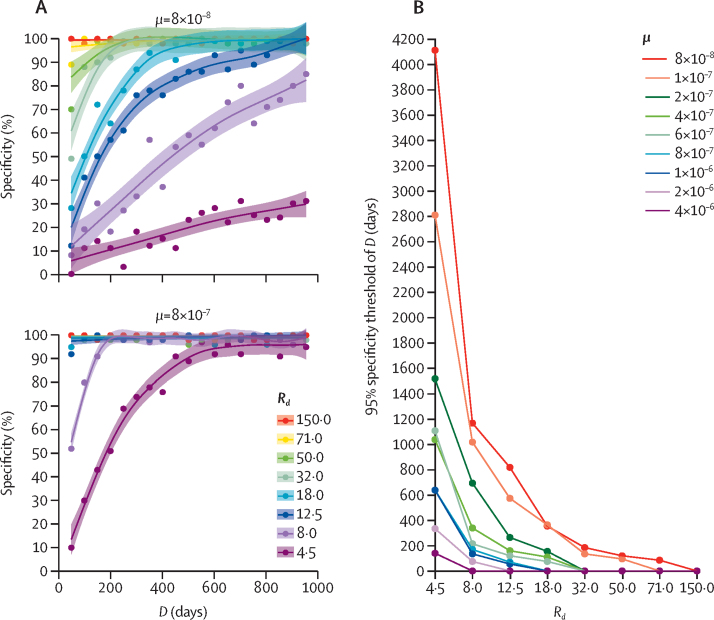
Assessment of the model's ability to classify outbreak isolates from the simulation study (A) Specificity of isolate classification when μ=8 × 10^−8^ or 8 × 10^−7^ substitutions per site per year. Each point provides a specificity value computed from 20 independent outbreaks simulated with the same input parameters, with *D* ranging from 50 to 1000 days (x-axis) and *R*_d_ varying between 4·5 and 150·0. (B) 95% specificity threshold value of *D* as a function of *R*_d_ (x-axis), computed for nine values of μ. *D*=duration of outbreak. *R*_d_=the ratio of evolution duration between non-outbreak and outbreak genomes. μ=number of substitutions per site per year.

To test the ability of the framework to distinguish between outbreak and non-outbreak cases, we generated independent synthetic outbreaks from different combinations of *D* and μ (appendix p 6). As expected, specificity was poor for lower values of μ, especially when the ratio of evolution duration between outbreak and non-outbreak genomes (*R*_d_) was small—ie, when non-outbreak genomes were more related genetically to outbreak genomes (figure 2A). By contrast, as expected, sensitivity was always high (>99%), irrespective of the parameter combinations (not shown; same parameters as figure 2A). We also observed that the 95% specificity *D*-value threshold decreased with increasing values of *R*_d_ and μ (figure 2B)—ie, less time is needed to accurately discriminate between outbreak and non-outbreak genomes when the non-outbreak genomes are more distinct or when the mutation rate is higher.

We next evaluated whether the model and framework could accurately estimate the parameters *D* and μ from outbreak data. To do so, we simulated synthetic outbreaks for which the values of *D* and *μ* were known, and attempted to estimate one or the other. Regarding *D* estimation, all 95% HPD estimates included the true value, and higher values of *D* were associated with smaller 95% HPD (appendix p 26). Similarly, μ was adequately estimated, and best estimates were closer to the target value for higher μ values (appendix p 26). Because higher *D* or μ values, or both, lead on average to more single-nucleotide polymorphisms (SNPs), greater precision in HPD estimates were expected in these cases.

We also investigated the effect of sampling density (ie, the number of isolates sampled divided by the outbreak duration in days) on estimation accuracy and precision (appendix p 27). First, increasing sampling density increases precision in the estimation of both *D* and μ, whatever the duration of the outbreak. Second, when the number of isolates is too low (<10), estimates are generally biased, with underestimation of both *D* and μ. This effect is shown on simulation results corresponding to the 60-day outbreaks (appendix p 27), when density is low (5–20%), which correspond to three to 12 isolates. Importantly, we show that sampling densities higher than 10% led to unbiased estimates.

**Figure 3:**
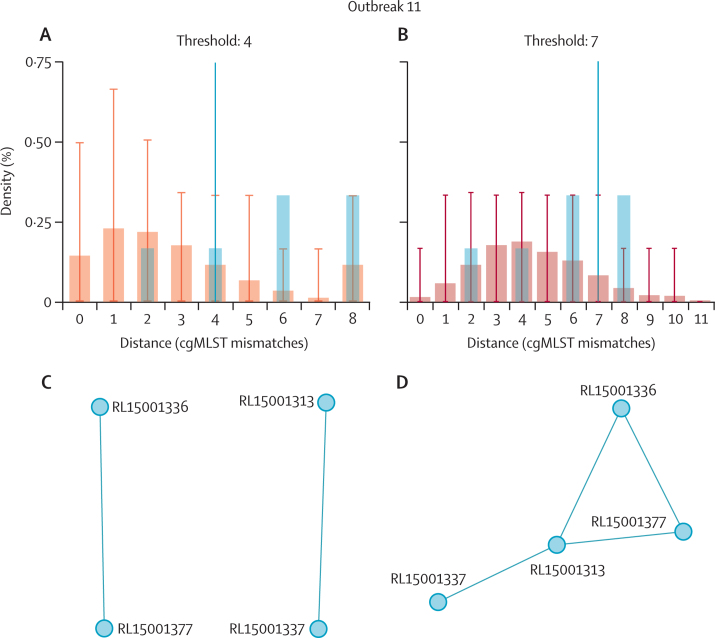
Distance threshold derived from the modelling framework, and its effect on clustering: example of outbreak 11 The core genome multilocus sequence typing distance distributions are shown with the observed distribution in blue (A and B), the simulated distribution without estimation in orange (A), and the simulated distribution using the estimated duration of the outbreak in red (B). Error bars represent the interval of prediction at 95% of 100 simulations. Dark blue vertical lines correspond to the derived distance threshold, defined here as the 99th percentile of the distributions from the observed distribution (A) and from the simulated distribution using the estimated duration of outbreak (B). Panels C and D show the single-linkage clusters resulting from the derived distance threshold corresponding to panels A and B, respectively. The isolates' names are taken from the original publication. The sameStrain process and coding for outbreak 11 is outlined in the appendix (pp 28–29). cgMLST=core genome multilocus sequence typing.

We finally used our framework to analyse data from outbreaks found in the literature. 16 outbreaks were included in our analysis (table),^7^, ^17^, ^18^, ^20^, ^21^, ^23^, ^25^ which are described in more detail in the appendix (pp 4–5). For each of the 16 identified published outbreaks, we applied our framework to estimate an expected outbreak-specific genetic threshold value (an example of outbreak 11 is shown in figure 3; all other outbreaks are shown in the appendix [pp 8–23]). We found that, for 14 of 16 outbreaks, the classification of isolates as being outbreak related or sporadic is consistent with previously reported proposals, stemming from epidemiological information. Four of these outbreaks included outliers (outbreaks 1, 4, 12, and 16), which were correctly classified as being beyond the threshold of exclusion estimated by our model, except for one isolate of outbreak 4 (table; appendix p 11; note that outbreak 4 comprised three different co-contaminating genetic clusters;^18^ here the defined outbreak strain was ST528). Ten other outbreaks (2, 3, 5, 6, 7, 9, 10, 13, 14, and 15) had no sporadic cases, and our framework clustered all previously suspected isolates as outbreak related.

**Table:** Analysis of 16 outbreaks from literature

	**Time period**	**Country**	**Bacteria**	**Sample size**		**Source**	**Genomic marker**	**Genome length (bp)**	**D_lit_**	**μ_lit_**	**Reference for μ_lit_**	**D_estimated_ (95% HPD)**	**μ_estimated_ (95% HPD)**	**Cutoff**		
				Human or animal	Food									Using D_lit_ and μ_lit_	Using D_estimated_	Using μ_estimated_
Outbreak 1; Octavia et al (2015)^7^	November, 2006	Australia	*Salmonella enterica* serovar Typhimurium	13	0	Chocolate mousse	SNP	4 857 450	120	12 × 10^−7^	Octavia et al (2015)^7^	95·64 (68·73 to 120·41)	1·01 × 10^−6^ (4·83 × 10^−7^ to 1·69 × 10^−6^)	8	7	7
Outbreak 2; Phillips et al (2014)^17^	January–May, 2014	Australia	*Salmonella enterica* serovar Typhimurium	25	0	Chicken liver pâté	SNP	4 857 450	20	12 × 10^−7^	Octavia et al (2015)^7^	20·51 (20·01 to 54·7)	6·33 × 10^−7^ (2·18 × 10^−7^ to 1·09 × 10^−6^)	1	1	1
Outbreak 3; Phillips et al (2014)^17^	January–May, 2014	Australia	*Salmonella enterica* serovar Typhimurium	20	0	Hot bread shop	SNP	4 857 450	38	12 × 10^−7^	Octavia et al (2015)^7^	44·92 (38·8 to 54·83)	1·13 × 10^−6^ (2·78 × 10^−7^ to 1·2 × 10^−6^)	2	2	2
Outbreak 4; Moffatt et al (2013)^18^	October– November, 2013	Australia	*Campylobacter jejuni*	7	2	Chicken liver pâté	SNP	1 343 000	9	3·23 × 10^−5^	Wilson et al (2009)^19^	28·08 (16·19 to 29·99)	1·38 × 10^−4^ (9·11 × 10^−5^ to 3·12 × 10^−4^)	4	11	11
Outbreak 5; Revez et al (2014)^20^	December, 2002–January, 2003	Finland	*Campylobacter jejuni*	4	2	Milk	cgMLST	1 432 000†	65	3·23 × 10^−5^	Wilson et al (2009)^19^	73·07 (66·26 to 95·73)	3·53 × 10^−5^ (2·19 × 10^−5^ to 3·07 × 10^−4^)	16	19	18
Outbreak 6; Grad et al (2012)^21^	May–July, 2011	Germany and France	*Escherichia coli O104:H4*	15	0	Sprouts	SNP	5 437 407	55	2·5 × 10^−6^	Grad et al (2013)^22^	107·41 (70·33 to 180·02)	5·39 × 10^−6^ (1·72 × 10^−6^ to 5·90 × 10^−6^)	7	13	13
Outbreak 7; Holmes et al (2015)^23^	2011	UK	*Escherichia coli O157*	10	0	Unwashed vegetables	SNP	4 122 236	340	2·26 × 10^−7^	Reeves et al (2011)^24^	352·83 (340·07 to 394·00)	3·21 × 10^−7^ (1·16 × 10^−7^ to 5·26 × 10^−7^)	2	2	2
Outbreak 8; Nielsen et al (2017)^25^	2012–13	B*	*Listeria monocytogenes*	5	10	Beef	cgMLST	1 462 000†	466	4·3 × 10^−7^	Halbedel et al (2018)^26^	494·49 (466·09 to 582·86)	2·09 × 10^−6^ (3·44 × 10^−7^ to 3·78 × 10^−6^)	2	2	7
Outbreak 9; Nielsen et al (2017)^25^	2007–13	B*	*Listeria monocytogenes*	5	3	Crab meat	cgMLST	1 732 000†	2200	4·3 × 10^−7^	Halbedel et al (2018)^26^	2270·80 (2200·54 to 2569·1)	4·83 × 10^−7^ (2·75 × 10^−7^ to 9·93 × 10^−7^)	12	12	13
Outbreak 10; Nielsen et al (2017)^25^	2013–14	B*	*Listeria monocytogenes*	5	4	Sandwiches	cgMLST	1 698 000†	289	4·3 × 10^−7^	Halbedel et al (2018)^26^	473·51 (318·76 to 499·83)	1·05 × 10^−6^ (3·78 × 10^−7^ to 3·63 × 10^−6^)	2	4	5
Outbreak 11; Nielsen et al (2017)^25^	2013–14	B*	*Listeria monocytogenes*	2	2	Ox tongue	cgMLST	1 464 000†	943	4·3 × 10^−7^	Halbedel et al (2018)^26^	1559·36 (1025·81 to 1599·92)	1·17 × 10^−6^ (6·22 × 10^−7^ to 4·10 × 10^−6^)	4	7	10
Outbreak 12; Nielsen et al (2017)^25^	2009–11	B*	*Listeria monocytogenes*	9	1	Unknown	cgMLST	1 685 000†	783	4·3 × 10^−7^	Halbedel et al (2018)^26^	805·65 (783·08 to 888·25)	2·46 × 10^−7^ (1·4 × 10^−7^ to 4·52 × 10^−7^)	6	6	4
Outbreak 13; Nielsen et al (2017)^25^	2013	T*	*Listeria monocytogenes*	4	1	Rakfisk	cgMLST	1 526 000†	180	4·3 × 10^−7^	Halbedel et al (2018)^26^	186·32 (76·94 to 299·33)	5·47 × 10^−7^ (8·25 × 10^−8^ to 2·98 × 10^−6^)	1	1	1
Outbreak 14; Nielsen et al (2017)^25^	2013–14	X*	*Listeria monocytogenes*	13	6	Foie gras	cgMLST	1 686 000†	161	4·3 × 10^−7^	Halbedel et al (2018)^26^	492·69 (234·34 to 499·94)	2·91 × 10^−6^ (1·1 × 10^−6^ to 4·29 × 10^−6^)	2	5	8
Outbreak 15; Nielsen et al (2017)^25^	2012	X*	*Listeria monocytogenes*	4	9	Cheese	cgMLST	1 698 000†	548	4·3 × 10^−7^	Halbedel et al (2018)^26^	384·79 (244·07 to 558·99)	3·54 × 10^−7^ (1·53 × 10^−7^ to 5·35 × 10^−7^)	5	4	4
Outbreak 16; Nielsen et al (2017)^25^	2012	C*	*Listeria monocytogenes*	25	0	Brie cheese	cgMLST	1 707 000†	150	4·3 × 10^−7^	Halbedel et al (2018)^26^	294·28 (252·25 to 299·99)	3·43 × 10^−6^ (1·81 × 10^−6^ to 4·3 × 10^−6^)	2	3	9

D_lit_ is the duration of the outbreak in days, as deduced from published literature, and μ_lit_ is the number of mutations per site per year found in the associated article or found elsewhere in the scientific literature (if unavailable in the associated article). For each μ_lit_ value, the reference value used is shown. *D*_estimated_ (in days) and μ_estimated_ were estimated based on three Markov Chain Monte Carlo chains; the associated 95% HPD for all outbreaks as well as the corresponding genetic threshold is shown. Each threshold results from the 99th percentile of a pairwise difference distribution from 100 outbreak simulations. cgMLST=core genome multilocus sequence typing. HPD=highest posterior density. SNP=single nucleotide polymorphism.

* Country code used in the reference article.

† Genome length is given by the number of loci *g* multiplied by the average gene length.

For two of the 16 outbreaks, conclusions from our model were discordant with published results. In outbreak 8 (*Listeria monocytogenes*, beef), two isolates were classified as outliers by our model (appendix p 11), whereas they were initially classified as outbreak related in the associated publication.^25^ In outbreak 11 (*L monocytogenes*, ox tongue), two isolates came from food and two others from humans. Our algorithm separated food samples into one cluster and human samples into another, whereas the isolates were initially grouped together based on epidemiological and genetic evidence.

When evaluating the influence of outliers on the inferred threshold by removing them from the analysis, we found that, in all cases, the absence of outliers did not affect the outbreak threshold. For outbreaks 1, 4, and 16, this removal did not change the threshold value, but improved the fit between the pairwise distance distribution from the observed data and from the simulated one (appendix p 7).

For each of the 16 outbreaks, we used our framework to re-estimate outbreak duration *D* (*D*_estimated_) and substitution rate μ (μ_estimated_) separately, and used these values (instead of *D*_lit_ and μ_lit_ taken directly from the literature and used above) to infer the genetic distance threshold (table).

For 10 of the 16 outbreaks, *D*_lit_ was well estimated: three *D*_lit_ values (outbreaks 1, 13, and 15) were within the corresponding HPD intervals and seven (outbreaks 2, 3, 5, 7, 8, 9, and 12) were just below. For the six remaining outbreaks, we found higher *D*_estimated_ values compared with previously reported *D*_lit_ (table). Regarding μ, for 11 outbreaks the HPD intervals included μ_lit_, whereas μ_estimated_ was lower than *μ*_lit_ for just one outbreak (outbreak 2), and higher than *μ*_lit_ for the four remaining outbreaks (outbreaks 4, 11, 14, and 16). It is important to note that for these four latter outbreaks, the 95% HPD of *D*_estimated_ was also higher than *D*_lit_ (table).

After re-analysing the outbreaks using our estimated values of either *D*_estimated_ or μ_estimated_ in lieu of *D*_lit_ or μ_lit_, we observed that the newly obtained thresholds did not affect the attribution of isolates to the outbreak or sporadic categories, with three exceptions. First, for outbreak 4, using *D*_estimated_ or μ_estimated_ increased the threshold from four to 11 SNPs, leading to the addition of the previously missing isolate but still excluding the outliers. Second, for outbreak 15, a decreased genetic threshold (four SNPs instead of five, in both independent estimation analyses for *D*_estimated_ and μ_estimated_) led to the exclusion of one isolate that was initially identified by the authors as belonging to the outbreak cluster. Finally, for outbreak 11, the genetic threshold was increased from four SNPs to seven SNPs using *D*_estimated_ and 10 SNPs using μ_estimated_, leading to the grouping of all isolates from both food and human samples (figure 3). We also observed that, in five of 16 cases, using *D*_estimated_ and μ_estimated_ improved the fit of the genetic distance distribution compared with *D*_lit_ or μ_lit_ (appendix p 7).

## Discussion

In this study, we developed an original evolutionary approach to the single-strain threshold conundrum that incorporates epidemiological and microbiological specificities of the outbreak under study. Our framework, which we have named sameStrain, had high sensitivity and specificity for isolate classification when tested using simulations and the results from 16 published datasets from real-world foodborne outbreaks. sameStrain led to consistent isolate classification for most of the 16 outbreaks, and refined the outbreak definition for two outbreaks.

Molecular surveillance facilitates the identification of common exposures to a single source of infection, even when dates and places of infection are distant.^27^, ^28^, ^29^ Given the high heterogeneity among the microbiological and epidemiological characteristics of different outbreaks, it is increasingly recognised that no universal single-species threshold exists that can be applied to distinguish between outbreak and non-outbreak isolates. This situation motivates a need for novel methods that estimate the expected genetic relatedness of outbreak isolates of a particular pathogen stemming from a common source, in the context of that outbreak's particular epidemiological characteristics. To our knowledge, Octavia and colleagues^7^ were the first to attempt to model the expected genetic distance among foodborne outbreak isolates. Although the authors incorporated mutation rate and outbreak duration in their model, they did not account for sampling dates. Consequently, their proposed thresholds depend on strong assumptions about the duration of the outbreak (referred to as the ex-vivo or in-vivo evolution time). Stimson and colleagues^12^ modelled the number of transmission events that separate infection cases, using a probabilistic model that incorporates the transmission process in addition to mutation rate and timing of infections. Because it models between-host transmission, this approach does not apply to point-source food outbreaks. Lastly, Coll and colleagues^14^ aimed at defining an SNP threshold above which transmission of *Staphylococcus aureus* between humans can be ruled out, by incorporating the timing of transmission and within-host diversity. This evolutionary modelling approach provides a robust SNP cutoff applicable to this specific ecological situation.

In our study, the simulation showed that our model performed well at grouping outbreak cases. We also observed that high values of *D* and μ led to more accurate estimates of genetic thresholds: in other words, model specificity increased with genetic diversity. This finding is akin to higher-resolution typing methods being better at discriminating related from non-related cases. We also found an effect of the evolutionary distance between outbreak and sporadic isolates on model specificity, consistent with known uncertainty in ruling out sporadic cases for genetically homogeneous pathogens. Additionally, we found that the sampling density is important, because it influences the number of observed genetic differences: outbreaks with low diversity will require more samples to capture enough pairwise differences for accurate estimation.

Our model assumes a constant pathogen population size *N* over time to avoid potential increases in computation time with growing populations. For accurate parameter estimation, the assumed value of *N* must be high enough to sufficiently capture the population's genetic diversity throughout the sampling process. Indeed, to increase its real-world applicability, our model simulates microbiological sampling processes and does not analyse the whole *N* population. Because λ, the Poisson parameter, is defined as a function of *N*, a population of 500 or 1000 individual bacteria is usually enough to capture all bacterial diversity, but higher values should be tested further when extreme substitution rates or durations are explored.

In most outbreak investigations, the time since initial source contamination is unknown, and underestimation of *D* is a common risk given the possibility of cryptic transmission—ie, unreported cases having occurred before initial outbreak detection.^30^ Previous knowledge of μ is also subject to uncertainty: this parameter strongly depends on the species, strain,^31^ environmental conditions (eg, temperature and cellular stress), and potentially other factors. Our results suggest that, although model-based estimates of *D* and μ were largely consistent with published information informed by epidemiological data collection, they were nonetheless often larger than previously assumed values from the literature. This finding suggests that assumed parameters were less consistent with the observed diversity, suggesting either a longer duration since source contamination or a faster effective substitution rate. As *D* and μ both affect the expected genetic diversity in the same direction, it is impossible to know whether it is the rate, or the duration, that was higher than initially suspected. We suggest that, in the absence of evidence for higher μ, fixing μ and estimating *D* might provide important clues regarding previous cryptic transmission. Considering higher *D* values than suggested by case recognition is clearly relevant for epidemiological investigations of outbreaks, because it widens the considered time window and might lead to the identification of initially unsuspected sources of contamination. When the sample size is high enough (eg, >10), re-estimation of these parameters is recommended to refine the analysis.

The analysis of the 16 published outbreaks led to the definition of genetic thresholds that were largely consistent with previous epidemiological evidence. For outbreaks 4 and 11, our model inferred a lower threshold than initially used in published reports of these outbreaks, defining as sporadic outliers some isolates that were initially considered as part of the cluster by the authors. When estimating the duration or substitution rate for both outbreaks, higher values were obtained by our model than values assumed from the literature. However, our model nonetheless grouped isolates consistently with respect to epidemiological evidence. Outbreak 11 involved foodborne listeriosis with contaminated food, where the two food samples differed by nine SNPs from the human samples, themselves separated by two SNPs. The two food samples were isolated from two food outlets that had the same meat producer. Because the incubation period of listeriosis is between 3 and 70 days, and because intermittent *L monocytogenes* contamination during meat production was observed,^32^ the duration of contamination *D* might have been higher than initially defined by the authors, suggesting that the true common ancestor of food and human isolates was in fact older than initially estimated in the original publication. This example illustrates how our estimation framework could inform epidemiological investigations. Interestingly, when using model-estimated duration of outbreak or substitution rate, we often observed an improved fit of the pairwise distance distributions (appendix p 7).

For outbreak 8, low quality of sequence data was observed for three genomes,^25^ including the two genomes excluded from the outbreak by our model. Low-quality data might have artificially inflated their genetic distinctness, which underlines the importance of input sequence data quality.

It is important to highlight the following limitations of our work. First, all presented results were generated by initialising the models with a fully homogeneous ancestral population. However, the contaminating population might be slightly heterogeneous if it has a non-negligible population size and had itself already evolved previously. In these cases, *D* might be interpreted as incorporating the diversification time before source contamination. Second, we only modelled mutation, neglecting other evolutionary processes such as genetic recombination. Detection of recombination among very closely related isolates is very challenging and its effect on genetic relatedness of co-occurring isolates would be negligible. However, recombination with genetically distinct co-contaminants might occur, and recombined chromosomal regions should be removed from the analysis, especially when using SNP-based analyses (by design, multilocus sequence typing moderates the effect of homologous recombination). Third, the model does not incorporate demographic events within the contaminated source, including population bottlenecks, which are potentially common in food-processing chains, but which would be challenging to infer and model. This limitation prevents the application of our model to outbreaks involving human-to-human transmission, be they community or hospital outbreaks. Finally, the framework is designed for a single evolving population derived from a single bacterial ancestor. However, when there is more than one contaminating genotype, our framework could be used for each of these separately.

We describe an innovative approach to the single-strain definition that uses genomic data and the most relevant epidemiological features of specific outbreaks to estimate an informed genetic distance threshold. This approach is grounded in evolutionary biology and alleviates the need for predefined thresholds, which are often not justified and might be inappropriate in most cases. The inferred, outbreak-specific genetic thresholds provide a reliable, non-arbitrary method of defining epidemiologically related cases of infection, and to exclude non-related sporadic isolates. This approach is fast and easy to use and can be run in real time, to generate an optimal threshold based on initial sampling dates, to rule out future samples. Upon subsequent source sampling or inclusion of suspected cases, it can be rerun for updated threshold definition (which is expected to increase with outbreak duration). The additional ability to estimate outbreak duration should also prove useful for common-source disease outbreak studies, by informing an appropriate temporal window for epidemiological investigations aimed at identifying and eliminating the source of contamination.

For **PulseNet** see https://www.cdc.gov/pulsenet/about/index.html

## Data sharing

All data and code used for this manuscript are available online at https://gitlab.pasteur.fr/BEBP/samestrain-r-package.

## Declaration of interests

We declare no competing interests.
