# Supplementary material for: Defining genomic epidemiology thresholds for common-source bacterial outbreaks: a modelling study
Source: Lancet Microbe. 2023 May;4(5):e349–57. doi: 10.1016/S2666-5247(22)00380-9 (PMC10156608; doi:10.1016/S2666-5247(22)00380-9)
Supplement: Supplementary appendix [file mmc1.pdf]

THE LANCET
Microbe

Supplementary appendix

This appendix formed part of the original submission and has been peer reviewed. We post it as supplied by the authors.

Supplement to: Duval A, Opatowski L, Brisse S. Defining genomic epidemiology thresholds for common-source bacterial outbreaks: a modelling study. *Lancet Microbe* 2023; published online March 29. https://doi.org/10.1016/S2666-5247(22)00380-9.

Supplementary appendix

Defining genomic epidemiology thresholds tailored to individual common-source bacterial outbreaks: a modelling study

Authors

Audrey Duval, PhD[1,2,3], Lulla Opatowski, PhD[1,2] and Sylvain Brisse, PhD[3]

Affiliations

[1]Epidemiology and modelling of bacterial escape to antimicrobials, Institut Pasteur, Paris, France.

[2]Anti-infective Evasion and Pharmacoepidemiology Team, CESP, Université Paris-Saclay, UVSQ, INSERM U1018, Montigny-le-Bretonneux, France.

[3] Institut Pasteur, Biodiversity and Epidemiology of Bacterial Pathogens, Paris, France

Contents

Supplementary text	pages 2-5
Supplementary tables	page 6-7
Supplementary figures	pages 8-27
Textbox panel	page 28
Supplementary references	page 30

Supplementary text

Methods

Evolutionary model and definition of the outbreak genetic distance threshold

Our evolutionary formalization is based on a Wright-Fisher forward model of haploid infectious agent evolution assuming a constant population size N. Here, we set N = 500, but any large-enough number from which samples will be drawn randomly would be suitable. Within our modelling framework, the five distinct properties characterising the infectious agent responsible for an outbreak are: i) *L*, the genome length (in base pairs, bp) or the average length of genes if gene-by-gene genotyping is used, such as in multilocus sequence typing (MLST) approaches; ii) *g*, the number of genes; *g* is equal to 1 when genome length is considered (when analysing core-genome SNP data, *L* should be set to the length of the core genome considered, rather than the whole genome length); iii) μ, the number of substitutions per site per year; iv) *D*, the duration (in days) of the outbreak (corresponding to the duration (in days) of (unfrozen) source contamination during which evolution and exposure of cases takes place, until the last recorded sample); and v) S_d, the set of sampling dates of isolates. Although *D* could potentially be approximated directly from S_d in some cases, it is useful to define *D* as being larger than the largest time interval within S_d, for example when suspicion exists that the source was contaminated much earlier than the first available sample. We define the end of the simulation process as the last sampling date, whilst the other sampling dates are defined backwards in time relative to their distance in time to the last sample.

The simulation is run for *D* days using one-day time steps, drawing the number of substitutions for the whole bacterial population at each time step. The evolutionary process is repeated for all time steps until *D* is reached. We assumed that the number of substitutions introduced at each time step during our simulation follows a Poisson distribution with parameter λ computed as:

$$\mu \sim \text{Poisson}\ (\lambda)$$

$$\lambda = \frac{\mu}{365}\, NLg \qquad (1)$$

where *N* is the population size, *L* is the length of the sequence (or the gene in MLST contexts) and *g* is the number of genes. A given individual can receive one or multiple substitutions at a given time step. All substitutions are considered unique (*i.e.*, lead to a new allele). At the end of this process, *n* individuals are randomly sampled to match the size of the studied dataset at sampling dates corresponding to those in the observed sample set *Sd*. A distribution of pairwise genetic distances is generated on these sampled individuals. We then chose the genetic threshold value as an upper percentile, *e.g.*, here the 99th percentile, of this distribution. If the genetic distance of an isolate to at least one other member of the group is less than or equal to the threshold value, this isolate is considered as a member of the outbreak group. Otherwise, the isolate is considered an outlier.

Estimation of the model's ability to discriminate between outbreak and outlier samples using simulated outbreaks

We applied our framework to a series of 171 simulated outbreaks generated with 19 different values of *D*, each combined with 9 values of μ and including simulated sporadic isolates. Synthetic datasets were built in two steps by generating, on the one hand, a series of samples corresponding to a simulated outbreak and, on the other hand, a series of samples corresponding to simulated outliers, here called sporadic isolates. These latter isolates were drawn from another simulated outbreak defined by $D_{sporadic}$; this approach enables the challenging of the model with varying degrees of distinctness between outbreak and sporadic isolates, with the latter resulting from distinct

durations of evolution since a common ancestor shared with the outbreak isolates. Details can be found in **Table S1**.

For a given analysis, we defined true positives (TP) as the real outbreak cases classified as a part of the outbreak, true negatives (TN) as sporadic cases classified as being excluded from the outbreak, false negatives (FN) as real outbreak cases excluded from the outbreak by the model, and false positives (FP) as the sporadic isolates misclassified as members of the outbreak. We then computed the sensitivity (*Se*) and specificity (*Sp*) of our approach based on the following formulae: $Se = \frac{TP}{TP+FN}$ and $Sp = \frac{TN}{FP+TN}$.

We set g = 1, L = 4,800,000 bp and N = 500. For each combination of values of D, μ and $D_{sporadic}$, 20 independent outbreaks with 20 samples and 5 sporadic cases outside the outbreak each were stochastically generated. For each outbreak, sample dates S_d were randomly selected as S_d ~ *Uniform*(0, D) and S_d ~*Uniform*($D_{sporadic}$-D, $D_{sporadic}$) for outbreak and sporadic isolates, respectively.

We defined R_d as the ratio of evolution duration of the sporadic population to the evolution duration of the outbreak population (*i.e.*, $R_d = \frac{D_{sporadic}}{D}$). To assess to which extent the genetic distance ratio between the outbreak and sporadic isolates can alter the quality of our results, we investigated the impact of varying the ratio of evolution duration R_d. Eight R_d values were explored: 4.5, 8, 12.5, 18, 32, 50, 71 and 150. For each R_d, 20 outbreak simulations were run, for all 171 distinct combinations of D and μ. In total, 27,360 outbreak-sporadic synthetic datasets were simulated.

For each combination of R_d and μ values, independent Generalized Additive Model (GAM) regressions were applied to investigate the link between specificity and duration. The minimum D required to guarantee 95% specificity was defined as the D-value corresponding to the 95% specificity threshold from the prediction of the GAM regression.

Estimation of the duration and substitution rate of outbreaks

To address uncertainty regarding the time since initial source contamination and evolutionary rate, we embed our model into a statistical framework to estimate either the duration of the outbreak (D) or its substitution rate (μ) (**Figure 1B**). We estimate D and μ from the observed pairwise genetic distance matrix by minimizing the distance described below, using a Markov Chain Monte Carlo (MCMC) approach with the Delayed Rejection Adaptive Metropolis (DRAM) algorithm to sample the model parameters (R package FME) [19]. For each possible set of parameters (L, g, S_d, D or/and μ), the least squares distance (LSD) between the observed pairwise genetic distance matrix and the expected one associated to the parameter set is calculated. For a given L, g, S_d, D and/or μ, the expected distribution of pairwise genetic distances is obtained by simulating population evolution using the sameStrain framework. Estimated parameters are provided by averaging over the best posterior estimates obtained for 3 independent MCMC chains and their corresponding 95% highest posterior density (HPD).
From the simulated distribution of pairwise genetic distances (for a given L, g, S_d, D and/or μ), the LSD is defined (under the 99th percentile, as the upper values are considered as non-outbreak related) as:

$$LSD = \sum_{i=1}^{n} (y_i - y'_i)^2 \qquad (2)$$

Where n is the highest difference in terms of the number of SNPs or cgMLST alleles under the 99th percentile between observed genomes y and simulated genomes y'.

Because of simulation stochasticity, for each MCMC iteration, 20 independent model simulations are run and averaged to build the expected distribution. In total, 10,000 iterations of the MCMC are run, with the 2000 first ones being discarded as a burn-in period. All MCMC chains are run with the modMCMC function from the FME package version 3.5.3 of R [19].

Validation. To assess the ability of the model to properly estimate D and μ, we run our framework on simulated outbreaks for which parameters were known. L, N and g were fixed as above. Outbreaks generated using combinations of (D, μ) were assessed, by varying durations over D = 60, 100, 200, 400 days and substitution rates over μ = 2E-07, 4E-07 and 6E-07 substitutions per site per year. Because the number of sampled genomes could affect estimation quality, and to assess to which extent this was the case, the number of samples was also varied in the analysis. For this, for each outbreak, 10 different sampling densities were evaluated, defined as the number proportion of sampled days, ranging from 5 to 50% of D with a step of 5 at each increment. For each combination of parameters, 20 individual outbreaks were run, leading to 2400 different simulated datasets. For each outbreak, we estimated D and μ separately with three independent MCMC chains.

Descriptions of published outbreaks investigated with the modelling framework

Outbreak 1 (1) was caused in November 2016 by *Salmonella enterica* serovar Typhimurium phage type DT170. The possible food source of contamination was chocolate mousse. Among 47 cases, 13 isolates were sequenced from patients. One isolate differed by 12 SNPs and another by five SNPs, which was interpreted as at least three distinct strains being present in the food source. We used the authors' high substitution rate and high duration of outbreak for D_{lit} and μ_{lit}.

Outbreaks 2 and 3 also involved *Salmonella* Typhimurium in Australia (2). These two outbreaks occurred between January and May 2014 in metropolitan Sydney. Outbreak 2 was related to a chicken liver pâté and outbreak 3 to a hot bread shop. At least 25 isolates for outbreak 2 and 20 isolates for outbreak 3 were sequenced and available (as well as some sporadic cases that we did not consider in the present paper). We used the same substitution rate for μ_{lit} as for Outbreak 1 and duration of outbreaks D_{lit} were chosen as the duration between the first and the last sample dates (+24H).

Outbreaks 4 (3) and 5 (4) involved *Campylobacter jejuni* infections caused by contaminated chicken liver pâté in Australia and milk in Finland, respectively. Outbreak 4 occurred from the end of October to the beginning of November 2013; nine isolates (seven from humans and two from food) were sequenced. Three distinct clusters were identified, ST528 (five isolates), ST535 (two isolates) and ST991 (two food isolates); here we focused on ST528 isolates. Outbreak 5 involved two *C. jejuni* isolates from a milk tank, two from humans and two from dairy cows, collected from December 2002 to January 2003. Substitution rates from Wilson et al. (5) were used for outbreaks 4 and 5 as μ_{lit}. We used the time difference between the first day of pâté preparation and the last swab day (+24H) for D_{lit} in outbreak 4. We used the duration between the first and the last sample dates (+24H) for D_{lit} in outbreak 5.

Outbreaks 6 (6) and 7 (7) were caused by *Escherichia coli* O104:H4 and O157, respectively. Outbreak 6 involved cases in Germany and France between May and July 2011, and caused bloody diarrhoea and haemolytic uremic syndromes. This outbreak was epidemiologically linked to contaminated sprouts. Four isolates were sequenced from German cases and 11 from French cases. In outbreak 7, *E. coli* O157 was linked to unwashed vegetables in the UK in 2011. A total of 10 isolates were available. Substitution rates from Grad et al. (8) and Reeves et al. (9) were chosen for μ_{lit}, respectively. For outbreak 6, the duration of outbreak D_{lit} was taken as the difference between first 55 days and last sample date because a traveller was suspected to be the cause of an introduction. For outbreak 7, we used the duration between the first and the last sample dates (+24H) for D_{lit}.

Finally, nine distinct outbreaks, labelled by us as outbreaks 8 to 16 (**Table 1**), were caused by *Listeria monocytogenes* (10). These outbreaks were linked to beef, crabmeat, sandwiches, ox tongue, an unknown source, rakfisk, foie gras, cheese and brie cheese, respectively, from 2011 to 2014 in four unknown countries referenced as B, T, X and C. Among the 10 isolates in outbreak 12, one was attributed to a separate clonal complex by MLST and another one (from milk) had no epidemiological evidence of being linked. In outbreak 16 from Brie cheese, the 25 isolates were a mix of outbreak and background isolates, with only 11 isolates ultimately being attributed to the outbreak. For these nine outbreaks, the substitution rate from Halbedel *et al*. (11) was used as μ_{lit}. For D_{lit}, the difference between first and last sample dates (+24H) was used for outbreaks 8, 9, 10, 11, 12 and 14, and we

added six months for outbreak 13 (time for fish ripening), four months and one month for outbreaks 15 and 16 (time for cheese ripening) and two months for outbreak 11.

Computational demands of our modelling framework

Execution time is highly dependent on model parameters. We assessed the execution on a computer with 32Go of RAM and a processor intel® cor™ i7-10610U CPU@ for four outbreaks; (i) outbreak 1 with classical D and μ, (ii) outbreak 6 with a shorter D and a larger μ, (iii) outbreak 10 with longer D and lower μ and (iv) outbreak 5 with a shorter D and the highest μ. Estimating the genetic threshold (with the classical 100 simulations) is very fast and takes around 0.1, 0.1, 0.2 and 1.6 seconds for outbreaks 1, 6, 10 and 5 respectively. Nevertheless, estimation of D or μ with one MCMC chain is much longer and takes around 40, 47, 100 and 1500 seconds for the estimation of μ as well as 46, 70, 43, and 300 seconds for the estimation of D for outbreaks 1, 6, 10 and 5, respectively. The higher were the values of D or μ, the longer the estimation was. Especially, μ had a more important impact on execution time.

Supplementary tables

Table S1. Simulations used to validate the model.

	**Number of *D* values used**	**Number of *μ* values used**	**Number of R_d values used**	**Number of sampling density values**	**Number of repetitions (per value set)**	**Total number of simulated outbreaks**
**Simulated outbreaks for threshold estimation**	19 (from 50 to 950 days)	9 (from 8E-08 to 4E-06 substitutions per site per year)	8 (4.5, 8, 12.5, 18, 32, 50, 71 and 150)	1 (20 outbreak samples and 5 outliers)	20	27 360
**Simulated outbreak for estimation of *μ* and *D***	4 (from 60 to 400 days)	3 (from 2E-07 to 6E-07)	0 (not relevant)	10 (from 5% to 50%, by increments of 5)	20	2 400

Table S2. Kolmogorov-Smirnoff (noted D_{KS}) test statistic for each of the 16 published outbreaks.

	D_{KS}		
**Outbreak**	D_{lit} **and** μ_{lit}	$D_{estimated}$	$\mu_{estimated}$
1	0.33	0.25	0.25
2	0.5	0.5	0.5
3	0.33	0.33	0.33
4	0.8	0.58	0.58
5	0.41	0.50	0.47
6	0.5	0.21	0.14
7	0.33	0.33	0.33
8	0.33	0.33	0.75
9	0.38	0.36	0.33
10	0.33	0.4	0.33
11	0.6	0.62	0.64
12	0.57	0.57	0.4
13	0.5	0.5	1
14	0.67	0.17	0.33
15	0.33	0.2	0.4
16	1	0.75	0.6

Outbreak numbers refer to Table 1. We analyzed the fit of the distribution of genetic distances obtained when using either D_{lit} and $\mu_{lit,}$ or $D_{estimated}$ or $\mu_{estimated}$, using the Kolmogorov-Smirnoff test statistic (D_{KS}). D_{KS} varies from 0 to 1. Small D_{KS} values indicate a better match of distributions. The use of MCMC-estimated values instead of those defined from the literature led to a better fit for outbreaks 1, 4, 6, 9 and 16 **(see also Supplementary Figures S1, S4, S6, S9, S16).** For outbreak 15, only $D_{estimated}$ improved the fit compared to both D_{lit} and μ_{lit} (**Supplementary Figure S10, S14 and S15**). For outbreak 12, a better fit was observed with $\mu_{estimated}$ compared to D_{lit} and μ_{lit} (**Supplementary Figures S1 and S12**). In contrast, the use of $\mu_{estimated}$ led to a worse fit for outbreaks 5, 8, 11 and 13 (**Supplementary Figures S5, S8, S11 and S13**). We note that the estimation of μ led to the addition of one isolate in outbreak 8, while it did not change the threshold of outbreak 13. For outbreak 8, the use of $\mu_{estimated}$ increased the threshold from 2 SNPs to 7 SNPs, while the fit of the genetic distance distribution was worse. Finally, no significant improvement or decline of the fit was observed for outbreaks 2, 3 and 7.

Supplementary figures

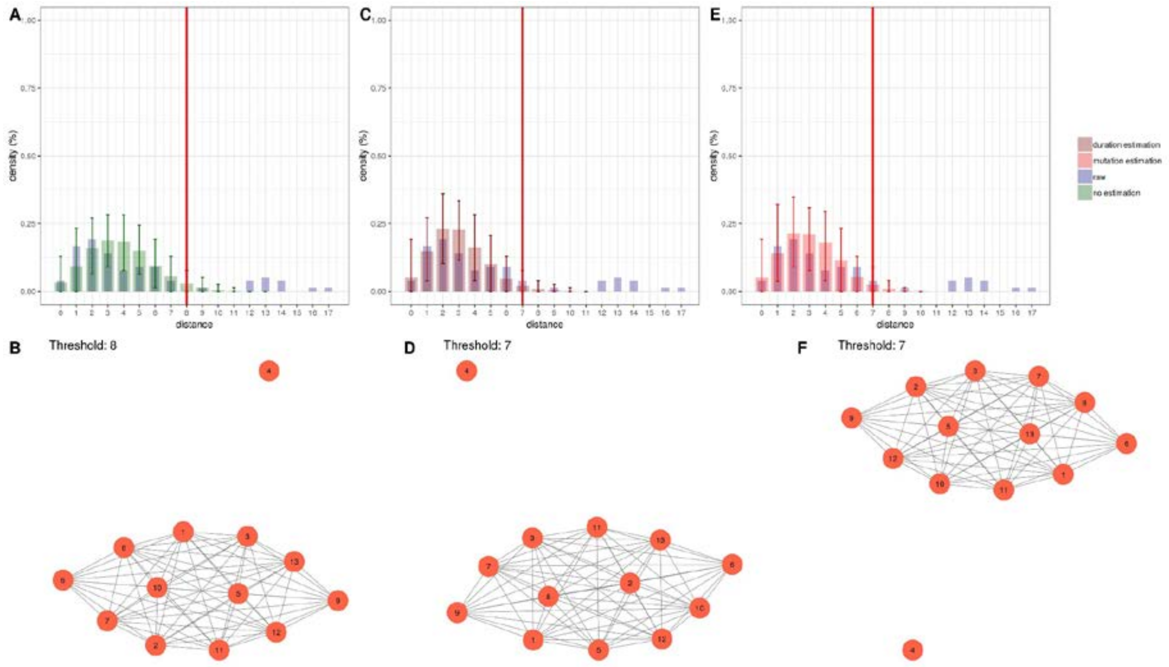

Figure S1. **Analysis of outbreak 1: Comparison between distance thresholds derived from the original publication on the outbreak, and from the current modelling framework.**

In A, C and E are presented the SNP distance distributions: observed distribution (blue), simulated distribution without estimation (green), simulated with the estimated duration of outbreak (dark red) and simulated with the estimated evolutionary rate (red). Error bars represent the interval of prediction at 95% of 100 simulations. Red vertical lines correspond to the derived distance threshold. In B, D and F are presented the resulting single-linkage clusters according to the derived distance threshold, defined here as the 99th percentile of the simulated distributions with values corresponding to panels A, C and E, respectively.

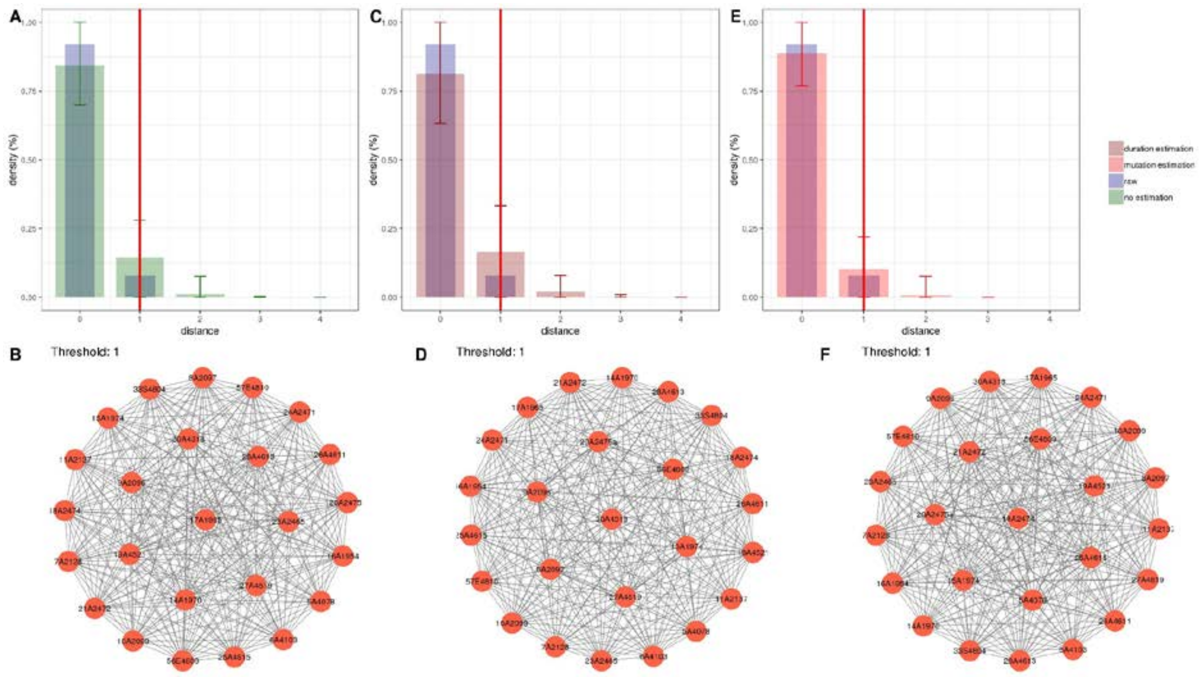

Figure S2. Analysis of outbreak 2: Comparison between distance thresholds derived from the original publication on the outbreak, and from the current modelling framework.

In A, C and E are presented the SNP distance distributions: observed distribution (blue), simulated distribution without estimation (green), simulated with the estimated duration of outbreak (dark red) and simulated with the estimated evolutionary rate (red). Error bars represent the interval of prediction at 95% of 100 simulations. Red vertical lines correspond to the derived distance threshold. In B, D and F are presented the resulting single-linkage clusters according to the derived distance threshold, defined here as the 99th percentile of the simulated distributions with values corresponding to panels A, C and E, respectively

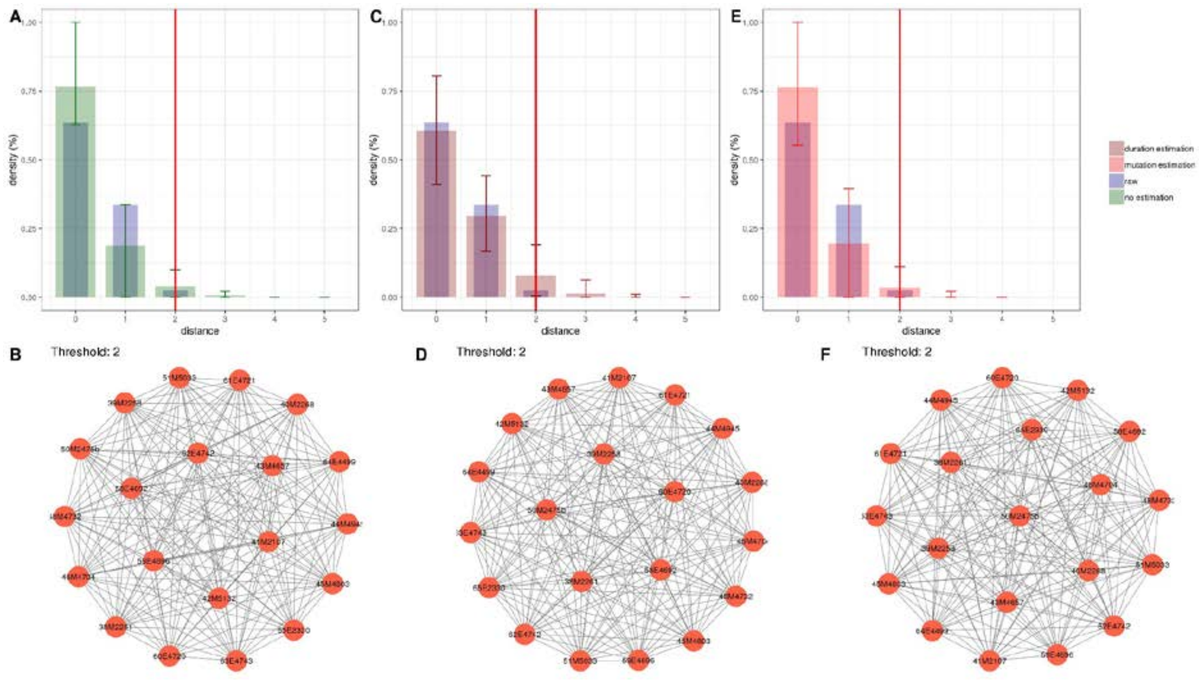

Figure S3. Analysis of outbreak 3: Comparison between distance thresholds derived from the original publication on the outbreak, and from the current modelling framework.

In A, C and E are presented the SNP distance distributions: observed distribution (blue), simulated distribution without estimation (green), simulated with the estimated duration of outbreak (dark red) and simulated with the estimated evolutionary rate (red). Error bars represent the interval of prediction at 95% of 100 simulations. Red vertical lines correspond to the derived distance threshold. In B, D and F are presented the resulting single-linkage clusters according to the derived distance threshold, defined here as the 99th percentile of the simulated distributions with values corresponding to panels A, C and E, respectively

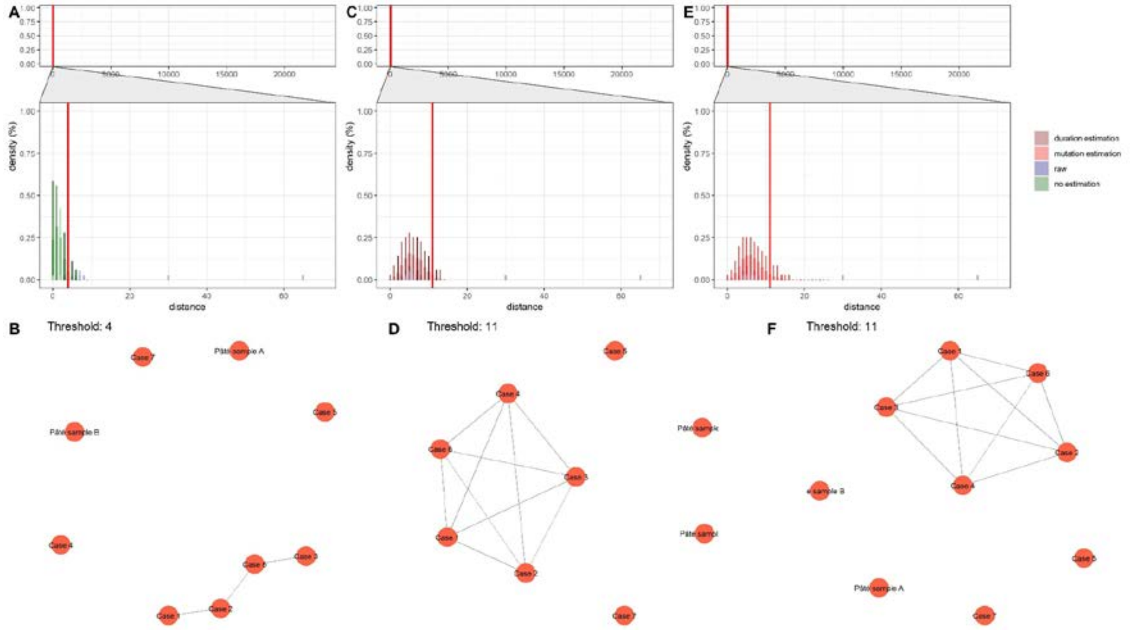

Figure S4. Analysis of outbreak 4: Comparison between distance thresholds derived from the original publication on the outbreak, and from the current modelling framework.

In A, C and E are presented the SNP distance distributions: observed distribution (blue), simulated distribution without estimation (green), simulated with the estimated duration of outbreak (dark red) and simulated with the estimated evolutionary rate (red). Error bars represent the interval of prediction at 95% of 100 simulations. Red vertical lines correspond to the derived distance threshold. In B, D and F are presented the resulting single-linkage clusters according to the derived distance threshold, defined here as the 99th percentile of the simulated distributions with values corresponding to panels A, C and E, respectively.

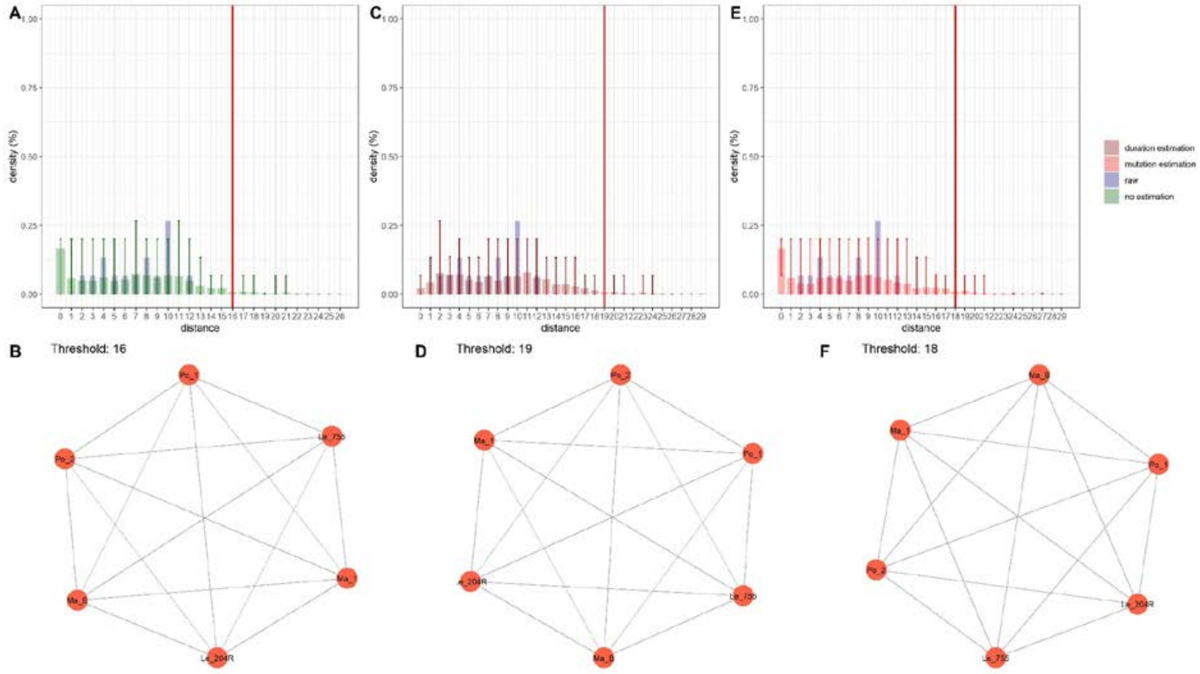

Figure S5. Analysis of outbreak 5: Comparison between distance thresholds derived from the original publication on the outbreak, and from the current modelling framework.

In A, C and E are presented the cgMLST distance distributions: observed distribution (blue), simulated distribution without estimation (green), simulated with the estimated duration of outbreak (dark red) and simulated with the estimated evolutionary rate (red). Error bars represent the interval of prediction at 95% of 100 simulations. Red vertical lines correspond to the derived distance threshold. In B, D and F are presented the resulting single-linkage clusters according to the derived distance threshold, defined here as the 99th percentile of the simulated distributions with values corresponding to panels A, C and E, respectively.

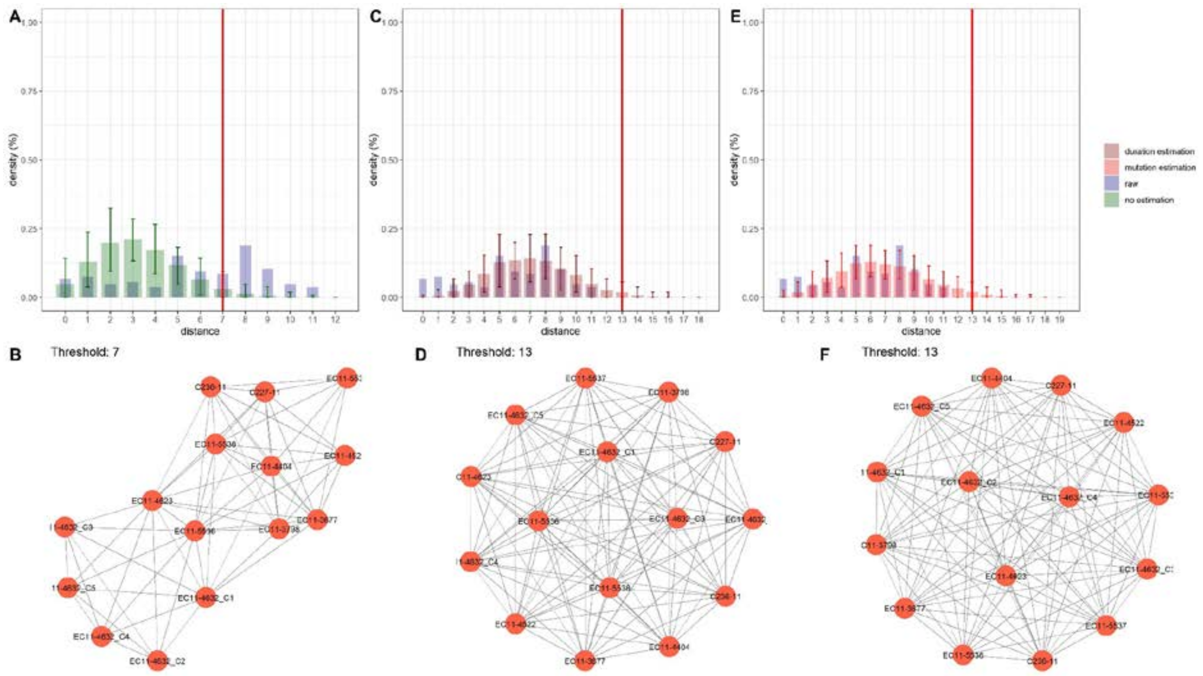

Figure S6. Analysis of outbreak 6: Comparison between distance thresholds derived from the original publication on the outbreak, and from the current modelling framework.

In A, C and E are presented the SNP distance distributions: observed distribution (blue), simulated distribution without estimation (green), simulated with the estimated duration of outbreak (dark red) and simulated with the estimated evolutionary rate (red). Error bars represent the interval of prediction at 95% of 100 simulations. Red vertical lines correspond to the derived distance threshold. In B, D and F are presented the resulting single-linkage clusters according to the derived distance threshold, defined here as the 99th percentile of the simulated distributions with values corresponding to panels A, C and E, respectively

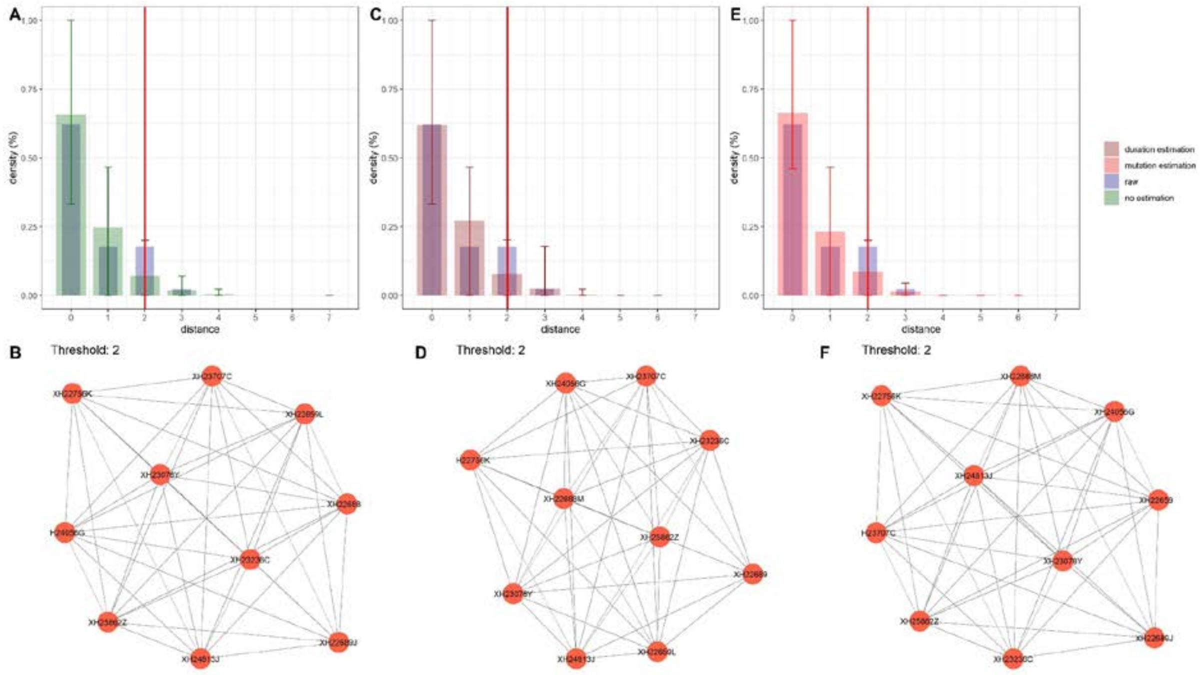

Figure S7. Analysis of outbreak 7: Comparison between distance thresholds derived from the original publication on the outbreak, and from the current modelling framework.

In A, C and E are presented the SNP distance distributions: observed distribution (blue), simulated distribution without estimation (green), simulated with the estimated duration of outbreak (dark red) and simulated with the estimated evolutionary rate (red). Error bars represent the interval of prediction at 95% of 100 simulations. Red vertical lines correspond to the derived distance threshold. In B, D and F are presented the resulting single-linkage clusters according to the derived distance threshold, defined here as the 99th percentile of the simulated distributions with values corresponding to panels A, C and E, respectively.

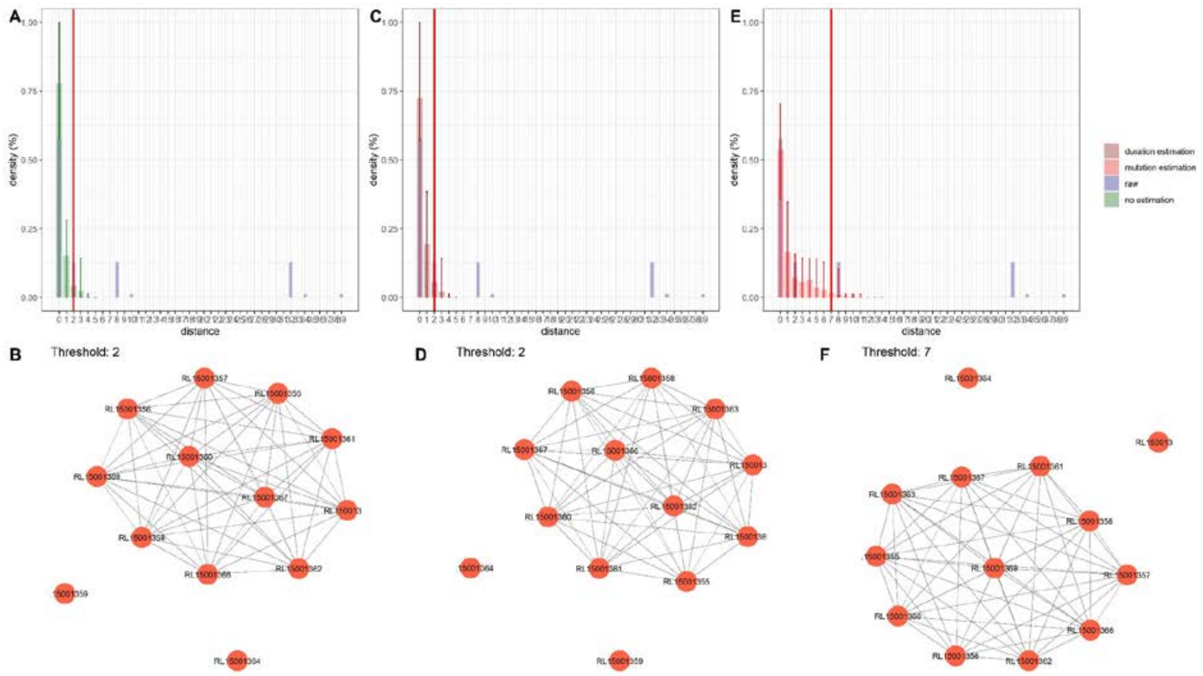

Figure S8. Analysis of outbreak 8: Comparison between distance thresholds derived from the original publication on the outbreak, and from the current modelling framework.

In A, C and E are presented the cgMLST distance distributions: observed distribution (blue), simulated distribution without estimation (green), simulated with the estimated duration of outbreak (dark red) and simulated with the estimated evolutionary rate (red). Error bars represent the interval of prediction at 95% of 100 simulations. Red vertical lines correspond to the derived distance threshold. In B, D and F are presented the resulting single-linkage clusters according to the derived distance threshold, defined here as the 99th percentile of the simulated distributions with values corresponding to panels A, C and E, respectively.

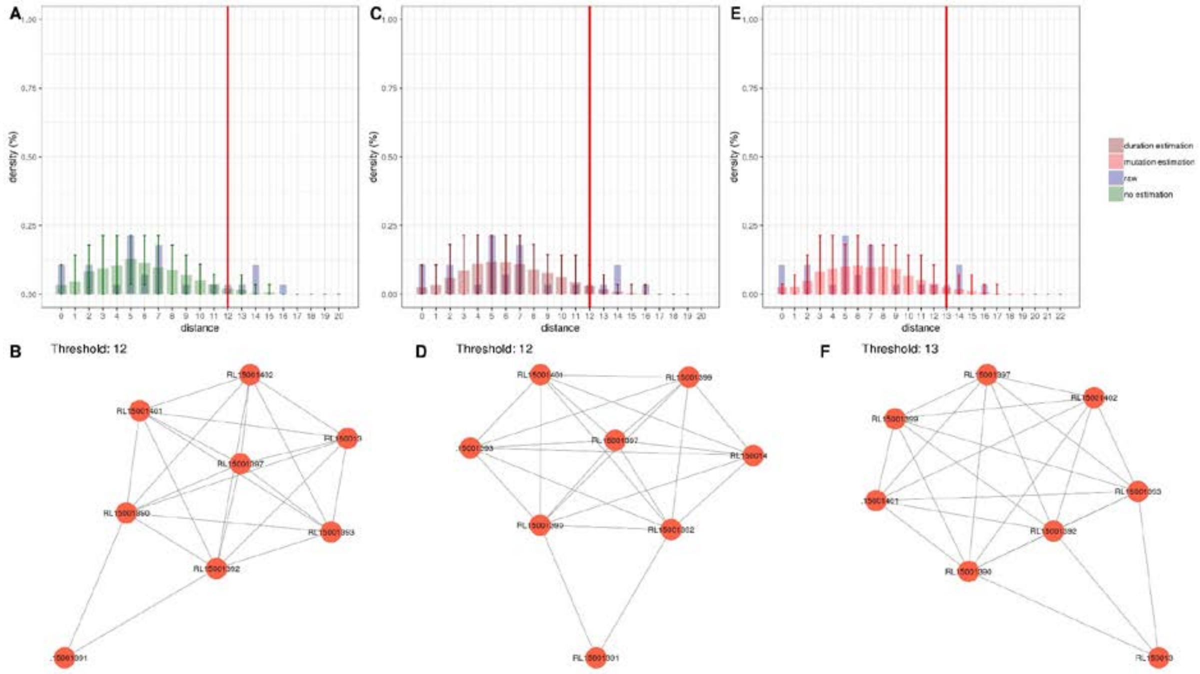

Figure S9. Analysis of outbreak 9: Comparison between distance thresholds derived from the original publication on the outbreak, and from the current modelling framework.

In A, C and E are presented the cgMLST distance distributions: observed distribution (blue), simulated distribution without estimation (green), simulated with the estimated duration of outbreak (dark red) and simulated with the estimated evolutionary rate (red). Error bars represent the interval of prediction at 95% of 100 simulations. Red vertical lines correspond to the derived distance threshold. In B, D and F are presented the resulting single-linkage clusters according to the derived distance threshold, defined here as the 99th percentile of the simulated distributions with values corresponding to panels A, C and E, respectively.

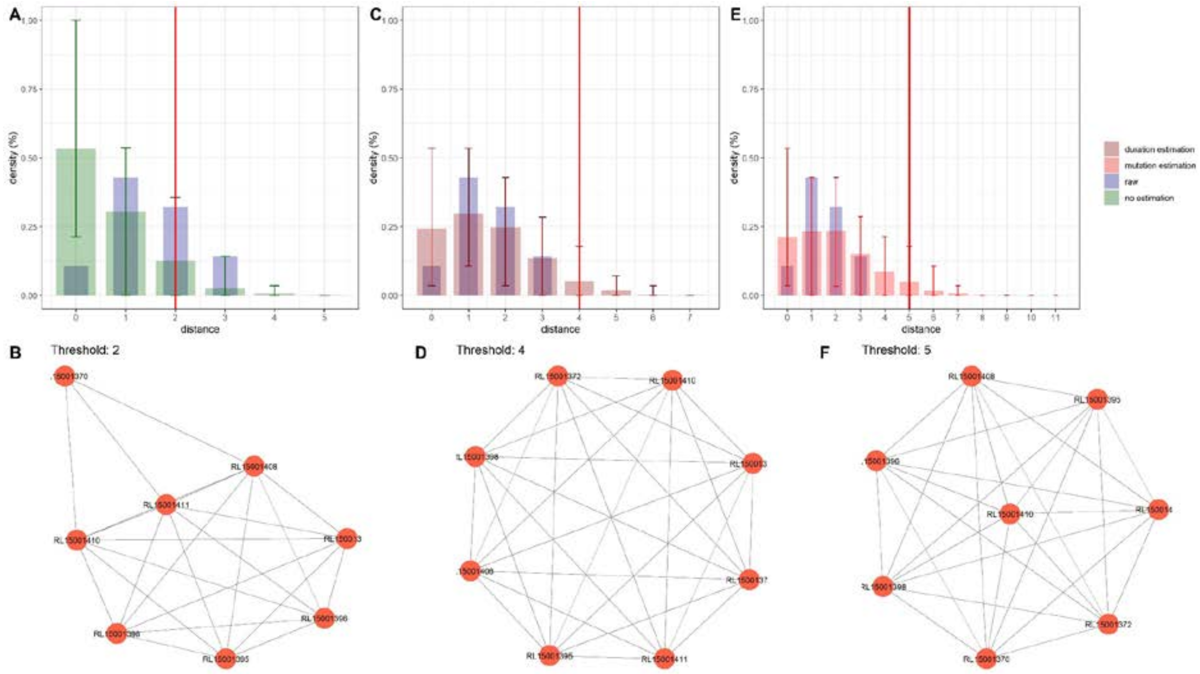

Figure S10. Analysis of outbreak 10: Comparison between distance thresholds derived from the original publication on the outbreak, and from the current modelling framework.

In A, C and E are presented the cgMLST distance distributions: observed distribution (blue), simulated distribution without estimation (green), simulated with the estimated duration of outbreak (dark red) and simulated with the estimated evolutionary rate (red). Error bars represent the interval of prediction at 95% of 100 simulations. Red vertical lines correspond to the derived distance threshold. In B, D and F are presented the resulting single-linkage clusters according to the derived distance threshold, defined here as the 99th percentile of the simulated distributions with values corresponding to panels A, C and E, respectively.

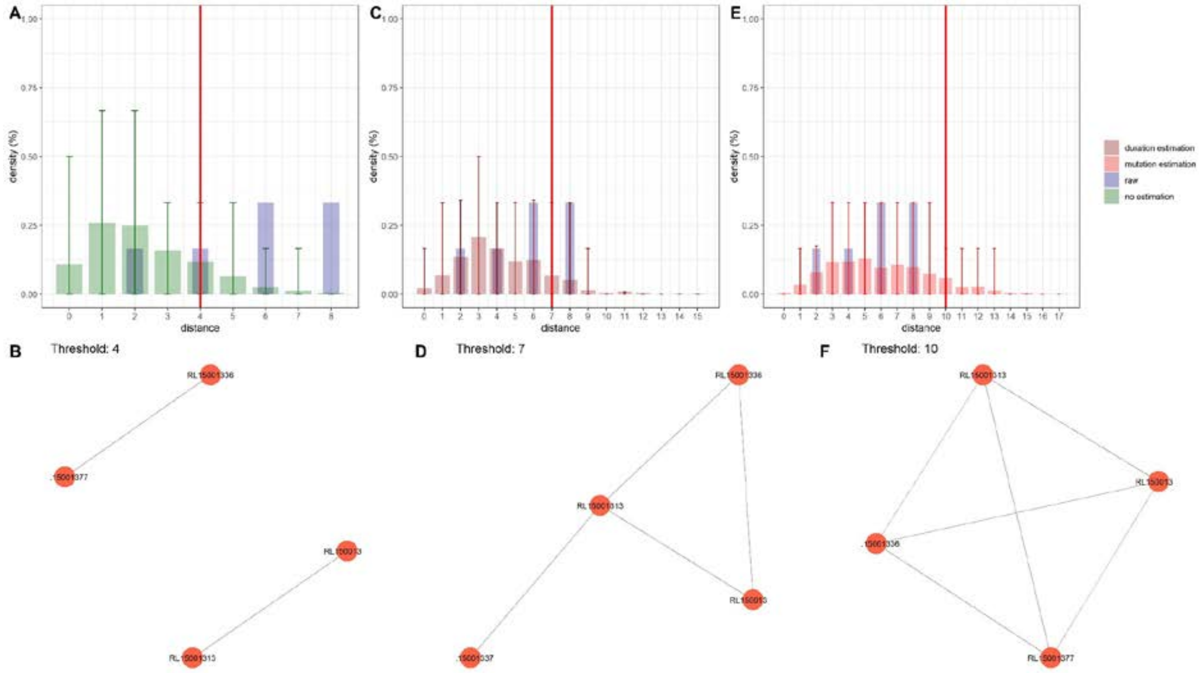

Figure S11. Analysis of outbreak 11: Comparison between distance thresholds derived from the original publication on the outbreak, and from the current modelling framework.

In A, C and E are presented the cgMLST distance distributions: observed distribution (blue), simulated distribution without estimation (green), simulated with the estimated duration of outbreak (dark red) and simulated with the estimated evolutionary rate (red). Error bars represent the interval of prediction at 95% of 100 simulations. Red vertical lines correspond to the derived distance threshold. In B, D and F are presented the resulting single-linkage clusters according to the derived distance threshold, defined here as the 99th percentile of the simulated distributions with values corresponding to panels A, C and E, respectively.

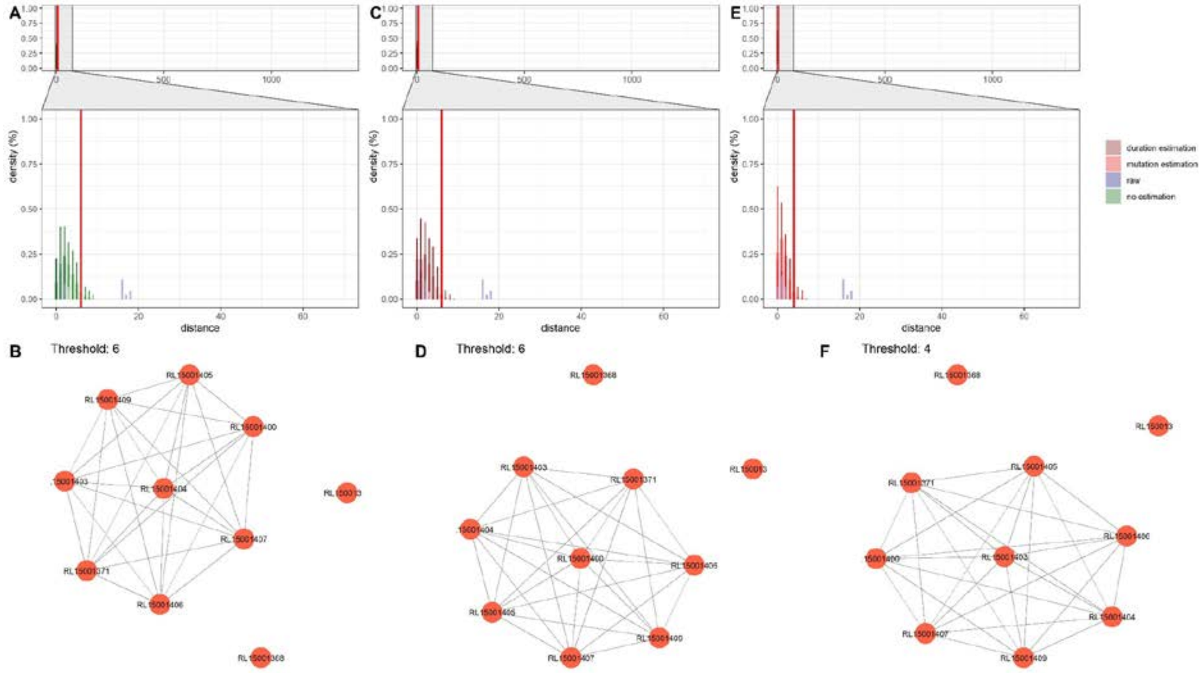

Figure S12. Analysis of outbreak 12: Comparison between distance thresholds derived from the original publication on the outbreak, and from the current modelling framework.

In A, C and E are presented the cgMLST distance distributions: observed distribution (blue), simulated distribution without estimation (green), simulated with the estimated duration of outbreak (dark red) and simulated with the estimated evolutionary rate (red). Error bars represent the interval of prediction at 95% of 100 simulations. Red vertical lines correspond to the derived distance threshold. In B, D and F are presented the resulting single-linkage clusters according to the derived distance threshold, defined here as the 99th percentile of the simulated distributions with values corresponding to panels A, C and E, respectively.

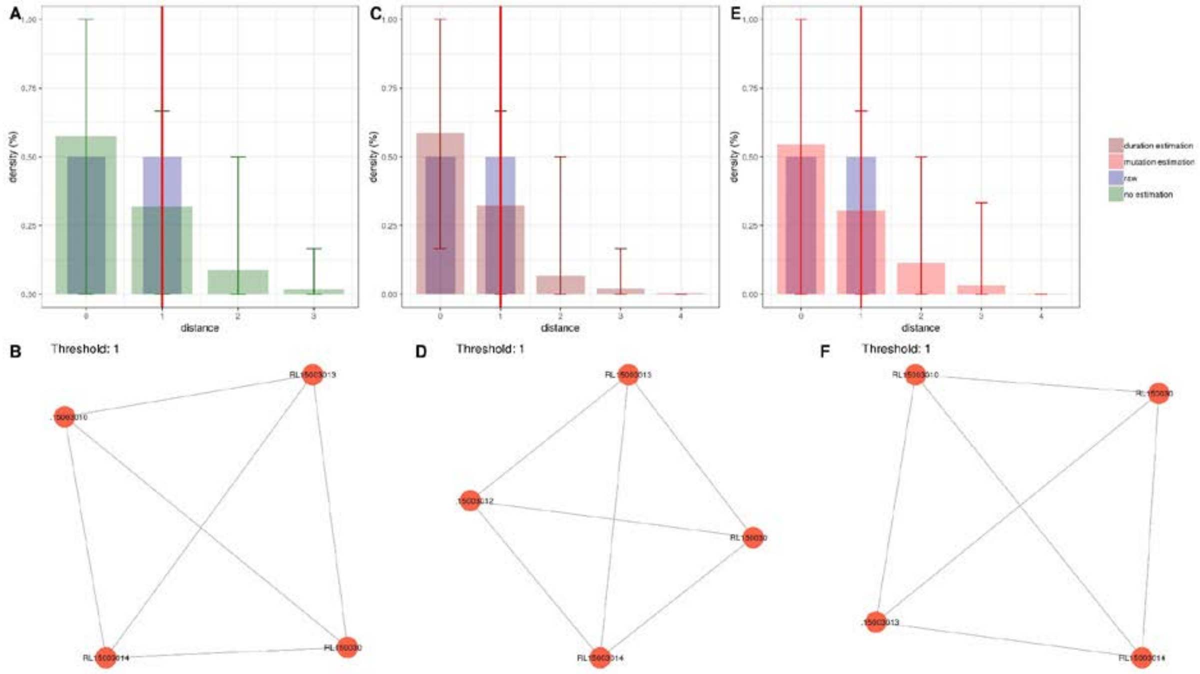

Figure S13. Analysis of outbreak 13: Comparison between distance thresholds derived from the original publication on the outbreak, and from the current modelling framework.

In A, C and E are presented the cgMLST distance distributions: observed distribution (blue), simulated distribution without estimation (green), simulated with the estimated duration of outbreak (dark red) and simulated with the estimated evolutionary rate (red). Error bars represent the interval of prediction at 95% of 100 simulations. Red vertical lines correspond to the derived distance threshold. In B, D and F are presented the resulting single-linkage clusters according to the derived distance threshold, defined here as the 99th percentile of the simulated distributions with values corresponding to panels A, C and E, respectively.

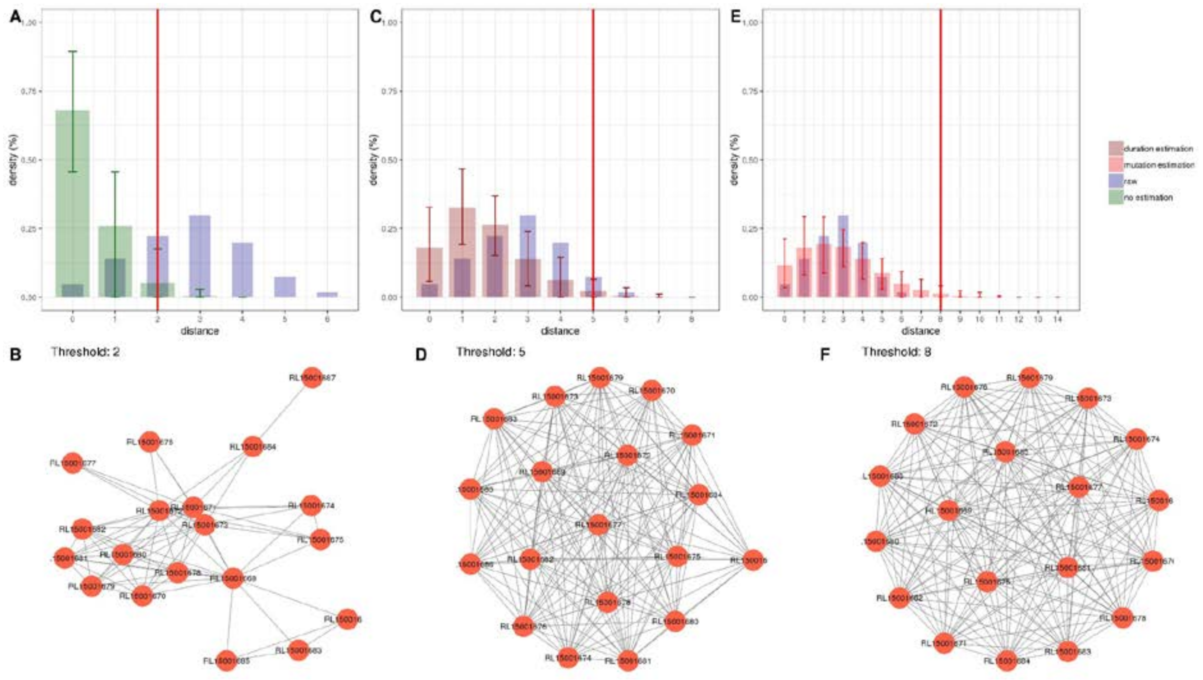

Figure S14. Analysis of outbreak 14: Comparison between distance thresholds derived from the original publication on the outbreak, and from the current modelling framework.

In A, C and E are presented the cgMLST distance distributions: observed distribution (blue), simulated distribution without estimation (green), simulated with the estimated duration of outbreak (dark red) and simulated with the estimated evolutionary rate (red). Error bars represent the interval of prediction at 95% of 100 simulations. Red vertical lines correspond to the derived distance threshold. In B, D and F are presented the resulting single-linkage clusters according to the derived distance threshold, defined here as the 99th percentile of the simulated distributions with values corresponding to panels A, C and E, respectively.

196

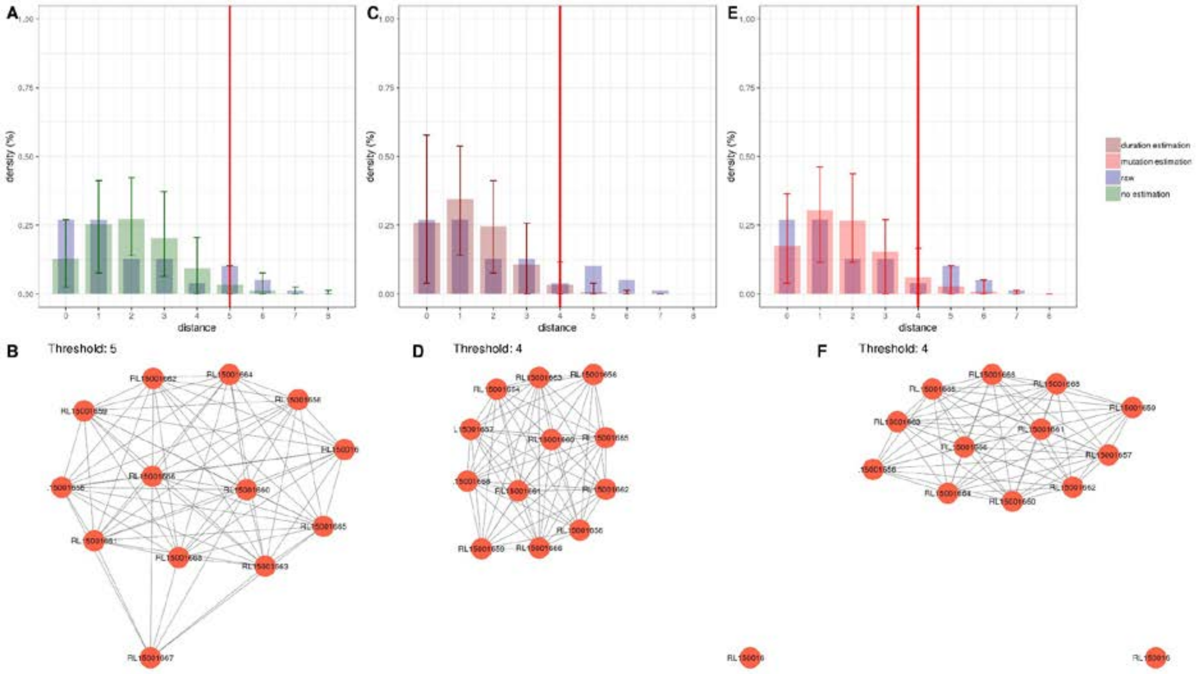

Figure S15. Analysis of outbreak 15: Comparison between distance thresholds derived from the original publication on the outbreak, and from the current modelling framework.

In A, C and E are presented the cgMLST distance distributions: observed distribution (blue), simulated distribution without estimation (green), simulated with the estimated duration of outbreak (dark red) and simulated with the estimated evolutionary rate (red). Error bars represent the interval of prediction at 95% of 100 simulations. Red vertical lines correspond to the derived distance threshold. In B, D and F are presented the resulting single-linkage clusters according to the derived distance threshold, defined here as the 99th percentile of the simulated distributions with values corresponding to panels A, C and E, respectively.

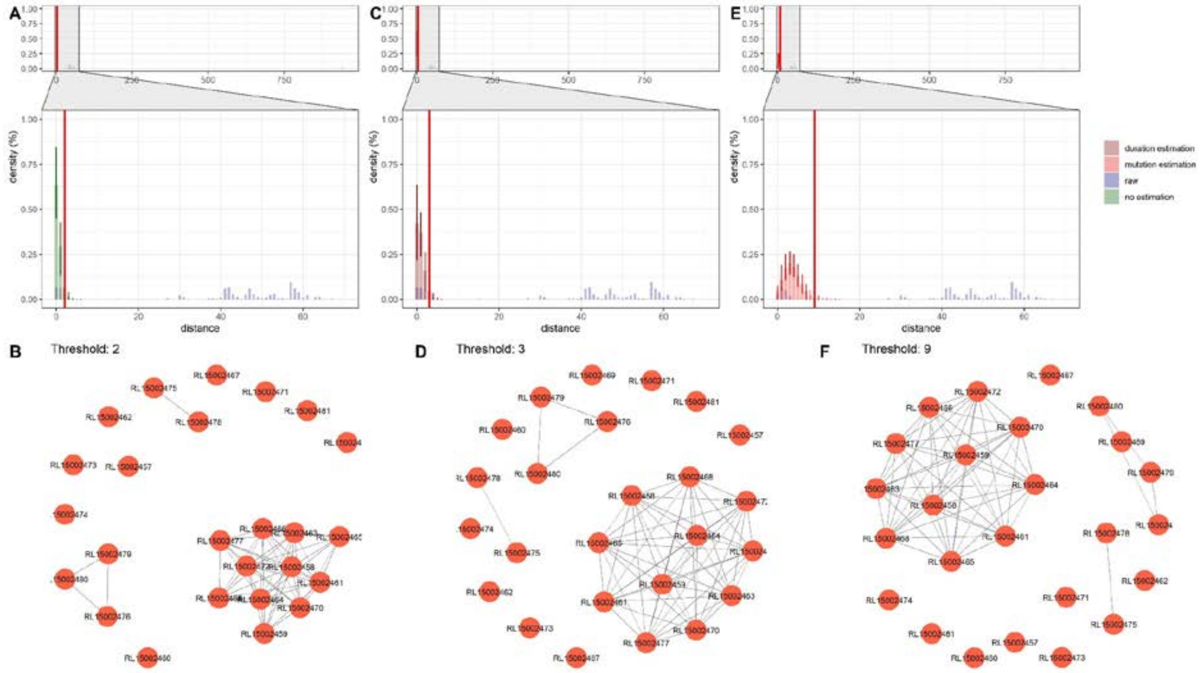

Figure S16. Analysis of outbreak 16: Comparison between distance thresholds derived from the original publication on the outbreak, and from the current modelling framework.

In A, C and E are presented the cgMLST distance distributions: observed distribution (blue), simulated distribution without estimation (green), simulated with the estimated duration of outbreak (dark red) and simulated with the estimated evolutionary rate (red). Error bars represent the interval of prediction at 95% of 100 simulations. Red vertical lines correspond to the derived distance threshold. In B, D and F are presented the resulting single-linkage clusters according to the derived distance threshold, defined here as the 99th percentile of the simulated distributions with values corresponding to panels A, C and E, respectively.

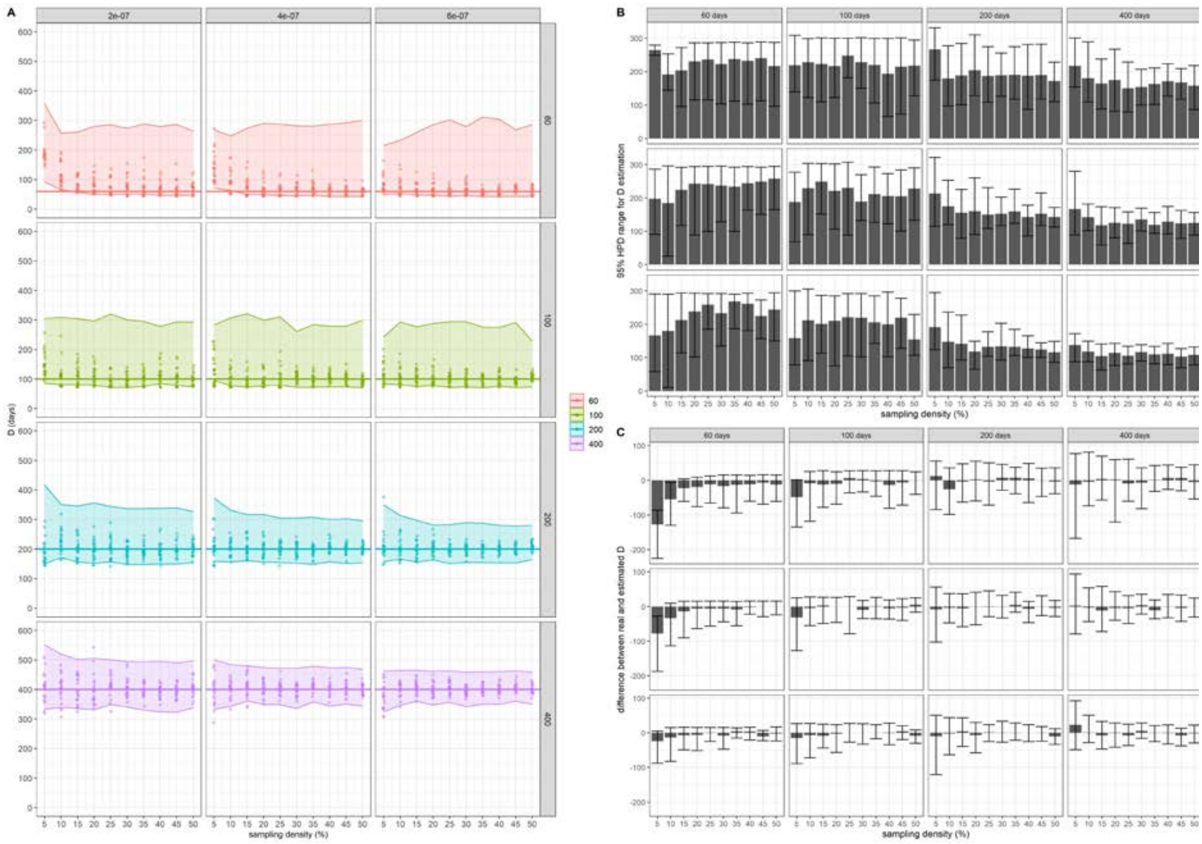

Figure S17. D estimation analysis. In A, average best estimation of *D* of the three MCMC chains for each of the 20 outbreaks. In B, average HPD range of the 20 outbreaks for 10 sampling densities (from 5% to 50%). In C, average distance between real and best estimated *D*. In B and C, error bars correspond to the 95% prediction interval.

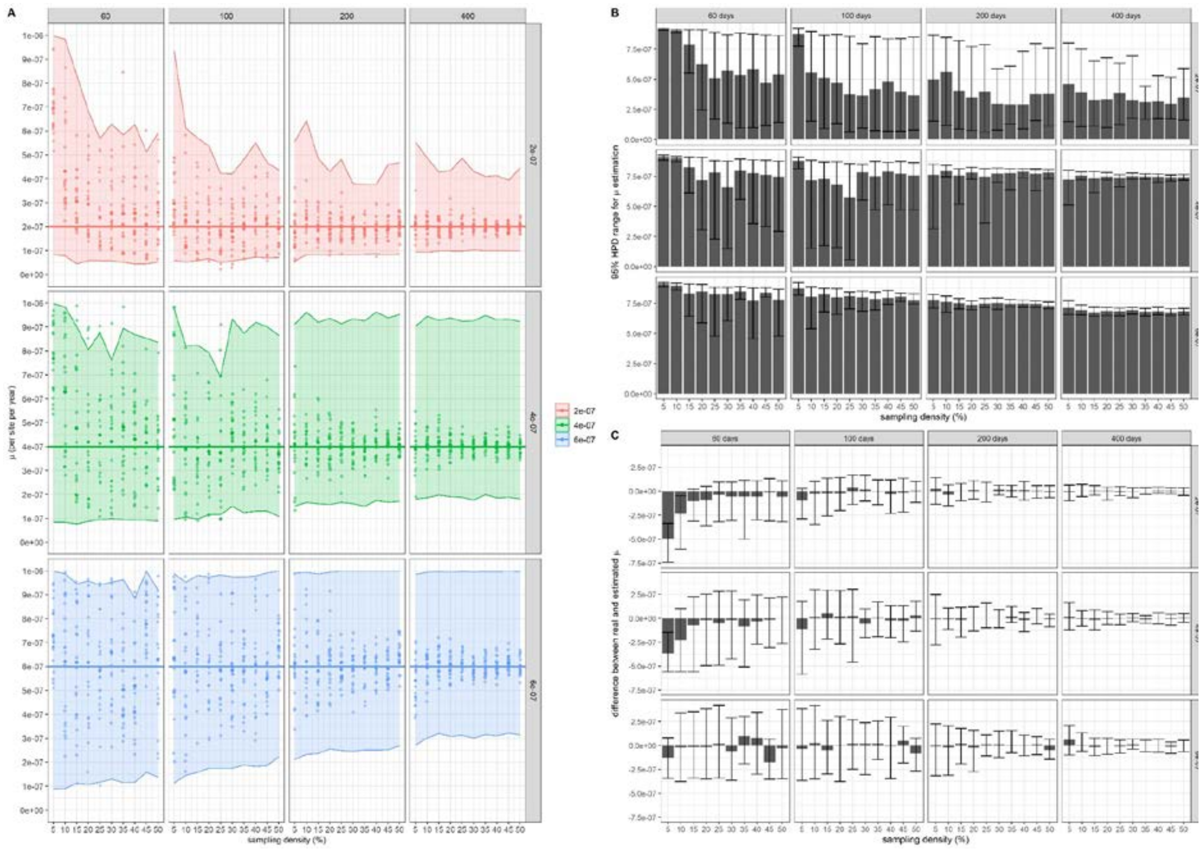

Figure S18. *μ* estimation analysis. In A, average best estimation of μ of the three MCMC chains for each of the 20 outbreaks. In B, average HPD range of the 20 outbreaks for 10 sampling densities (5 to 50%). In C, average distance between real and best estimated μ. In B and C, error bars correspond to the 95% prediction interval.

Figure S19. Assessment of the quality of the estimation of *D* and *μ* through simulation.

Precision of the estimation of *D* (**A**) and *μ* (**B**) from simulated data generated using different values of *D* and *μ*. The sample size, defined as the number of observed samples and associated dates, was set to 0.2x*D*. On each panel, the red line indicates the expected *D* (A) and *μ* (B) values used to generate the simulated data (the precise value is indicated above each panel). For each of the 240 synthetic outbreaks analysed, three independent MCMC chains were run, and the three corresponding best estimates are shown (points). Vertical bars represent the average values of the minimum and maximum of the 95% credibility interval of the 3 MCMC chains. Each colour corresponds to distinct values of *μ* or *D* used in the simulations (see keys).

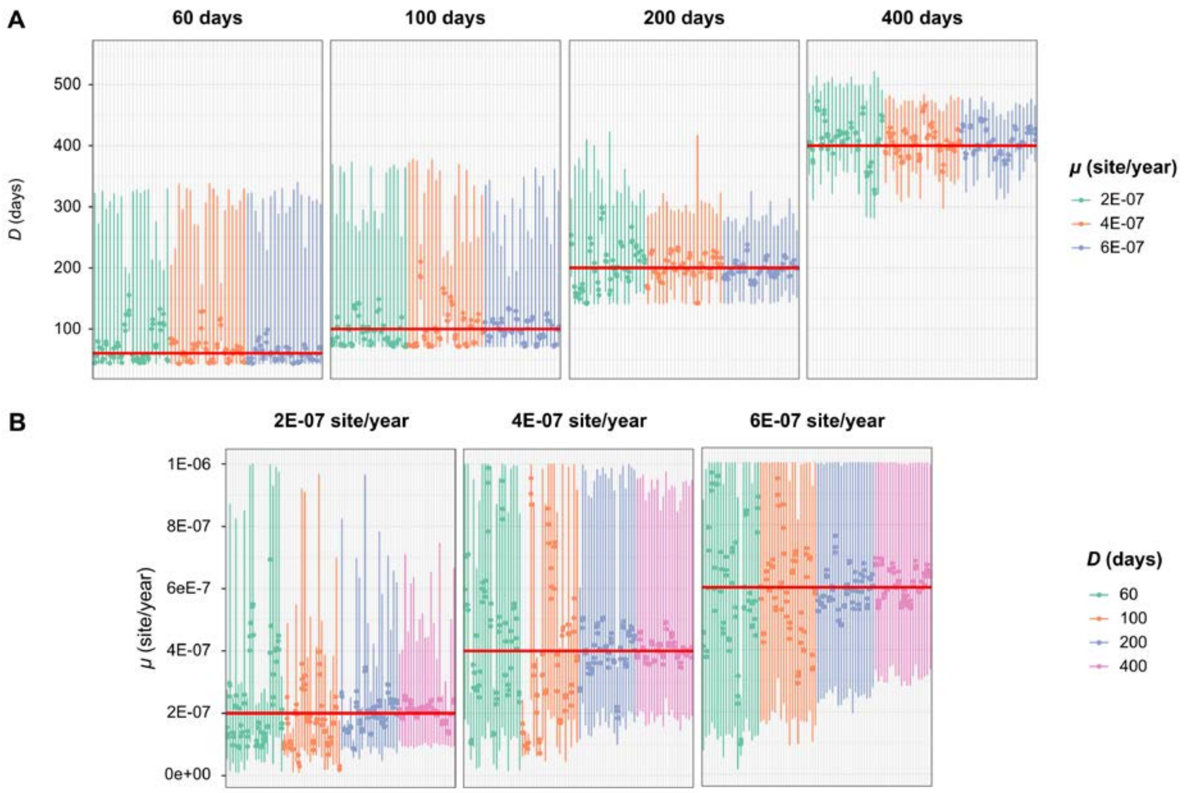

Figure S20. Impact of sampling density on the precision of the estimation of *D* and *μ*.

Sampling density represents the number of isolates sampled, divided by the outbreak duration in days (expressed as a percentage, roughly corresponding to the percentage of days at which a sample was drawn; sampling of days at which an isolate was drawn was performed with replacement). The position of each symbol represents the difference between the expected and best estimates of *D* (A) and *μ* (B) for each of 2400 outbreaks simulated using combinations of 4 values of *D* (60, 100, 200 and 400 days; represented in the four rows) and 3 values of *μ* (2E-07: blue diamonds, 4E-07: orange circles, 6E-07, red triangles; values in substitutions per site per year; see key).

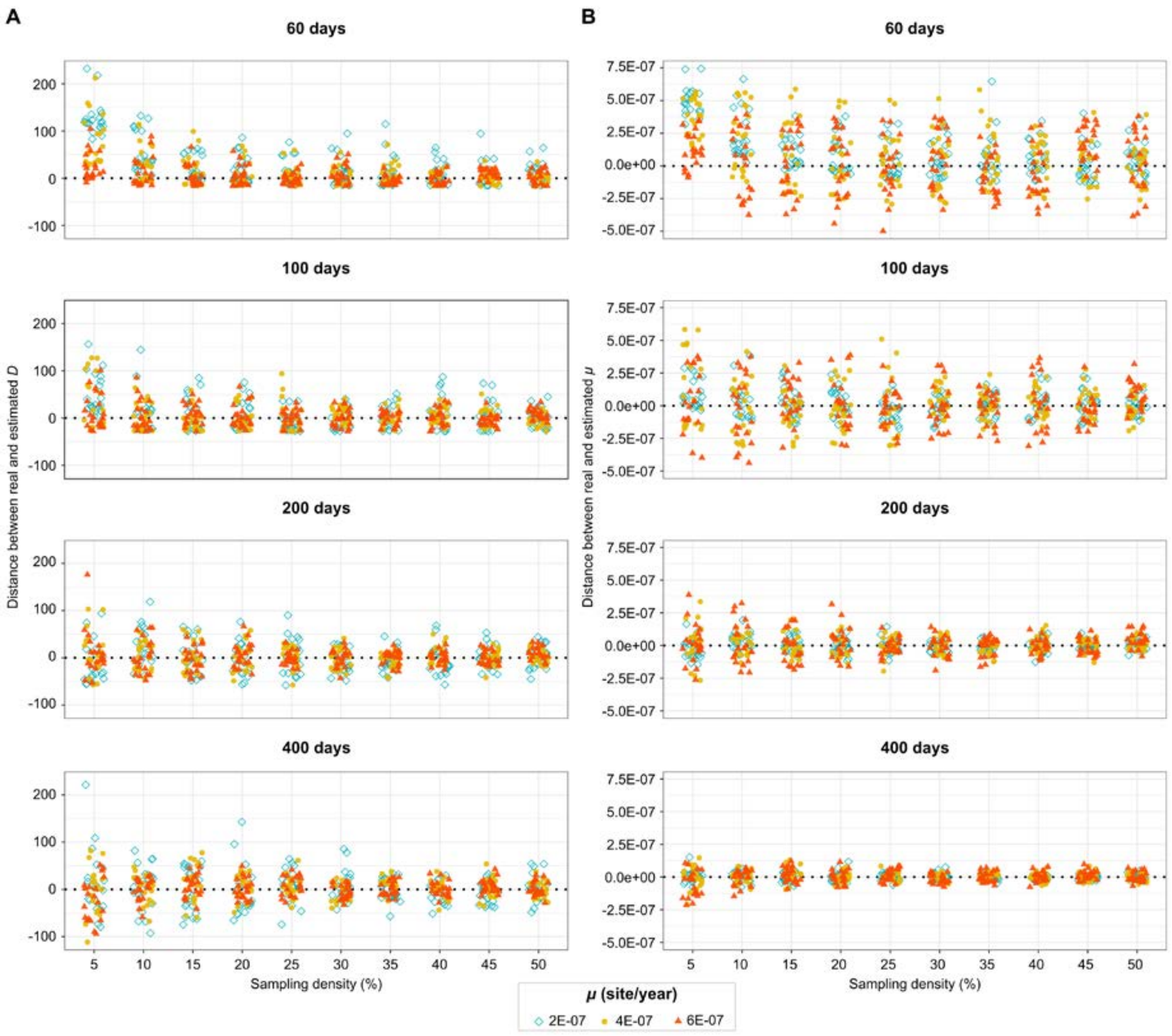

Textbox panel: use of the SameStrain framework, taking outbreak 11 as an example

We here provide an example of use of the SameStrain framework, taking outbreak 11 as an example. Outbreak 11 corresponds to outbreak 4 in Nielsen *et al*.; and was originally described in Lamden *et al.* 2013. There were two human cases separated by two years' time; and two isolates from ox tongue samples separated by at least 6 months. We use outbreak duration Dlit = 943 days (2 years plus 7 months). Whole genome sequencing showed that the two human types are almost identical (2 cgMLST mismatches) whereas the food isolates are 6-8 cgMLST alleles distant. The cgMLST scheme has 1685 genes considered of average size 1000 nt. The substitution rate Mu_{lit} is set to 4.3 E-07 (Halbedel *et al.*, 2018; ref 35).

1. To define the outbreak threshold, the above parameter values were used:

```
gene.length = 1000
n.gene = 1685
duration = 943
mutation.user = 0.00000043
type="MLST"
step="day"
n.isolat = 500
```

and the simulation was run:

```
SIM<-simulEvolution(dates, n.isolat, n.gene, gene.length, mutation.user, duration, type, step, nsim)
```

with dates :

```
##          ID      dates
## 4 RL15001377 2009-03-26
## 1 RL15001313 2011-04-12
## 2 RL15001336 2011-10-05
## 3 RL15001337 2011-10-24
```

which outputs threshold T = 4 (**Figure 3A**) based on the 99th percentile

Next, using the observed genetic distance matrix ('snp' parameter in the following command line):

```
##            RL15001313 RL15001336 RL15001337 RL15001377
## RL15001313          0          6          4          6
## RL15001336          0          0          8          2
## RL15001337          0          0          0          8
## RL15001377          0          0          0          0
```

we also plot the observed distribution (**Figure 3A**) using the command :

```
plotDistribution(SIM, snp, T)
```

2. Estimating the duration or substitution rate using the framework:

Here, we provide the case where we set the substitution rate as Mu_{lit}, and estimate the outbreak duration; the MCMC chain is run in the following way :

```
pMin = 943
pMax = 1600
pMiddle = 1000
all_parms <- c(pMiddle, pMin, pMax)
limit = round(as.numeric(findPercentileThreshold(snp, 0.99)))
MCMC = simulPopBMCMC(all_parms, dates, snp, n.isolat, n.gene, gene.length, mutation.user, duration, typ
e, step="day", n.iter=1000, burnin=20, chain=3, toEstimate="duration", returnCompleteResult=TRUE)
```

3. Using the estimated duration (1480 days), the simulation is then run again:

```
SIM<-simulEvolution(dates, n.isolat, n.gene, gene.length, mutation.user, duration_estimation, type, ste
p, nsim)
```

leading to threshold T = 7 cgMLST mismatches (**Figure 3B**)

Supplementary references

1. Octavia S, Wang Q, Tanaka MM, Kaur S, Sintchenko V, Lan R. Delineating Community Outbreaks of Salmonella enterica Serovar Typhimurium by Use of Whole-Genome Sequencing: Insights into Genomic Variability within an Outbreak. J Clin Microbiol. avr 2015;53(4):1063-71.

2. Phillips A, Sotomayor C, Wang Q, Holmes N, Furlong C, Ward K, et al. Whole genome sequencing of Salmonella Typhimurium illuminates distinct outbreaks caused by an endemic multi-locus variable number tandem repeat analysis type in Australia, 2014. BMC Microbiol. 15 2016;16:211.

3. Moffatt CRM, Greig A, Valcanis M, Gao W, Seemann T, Howden BP, et al. A large outbreak of Campylobacter jejuni infection in a university college caused by chicken liver pâté, Australia, 2013. Epidemiol Infect. oct 2016;144(14):2971-8.

4. Revez J, Zhang J, Schott T, Kivistö R, Rossi M, Hänninen M-L. Genomic variation between Campylobacter jejuni isolates associated with milk-borne-disease outbreaks. J Clin Microbiol. août 2014;52(8):2782-6.

5. Wilson DJ, Gabriel E, Leatherbarrow AJH, Cheesbrough J, Gee S, Bolton E, et al. Rapid Evolution and the Importance of Recombination to the Gastroenteric Pathogen Campylobacter jejuni. Mol Biol Evol. févr 2009;26(2):385-97.

6. Grad YH, Lipsitch M, Feldgarden M, Arachchi HM, Cerqueira GC, Fitzgerald M, et al. Genomic epidemiology of the Escherichia coli O104:H4 outbreaks in Europe, 2011. Proc Natl Acad Sci U S A. 21 févr 2012;109(8):3065-70.

7. Holmes A, Allison L, Ward M, Dallman TJ, Clark R, Fawkes A, et al. Utility of Whole-Genome Sequencing of Escherichia coli O157 for Outbreak Detection and Epidemiological Surveillance. J Clin Microbiol. nov 2015;53(11):3565-73.

8. Grad YH, Godfrey P, Cerquiera GC, Mariani-Kurkdjian P, Gouali M, Bingen E, et al. Comparative Genomics of Recent Shiga Toxin-Producing Escherichia coli O104:H4: Short-Term Evolution of an Emerging Pathogen. mBio [Internet]. 1 mars 2013 [cité 19 juin 2020];4(1). Disponible sur: https://mbio.asm.org/content/4/1/e00452-12

9. Reeves PR, Liu B, Zhou Z, Li D, Guo D, Ren Y, et al. Rates of Mutation and Host Transmission for an Escherichia coli Clone over 3 Years. PLoS ONE [Internet]. 27 oct 2011 [cité 17 juin 2020];6(10). Disponible sur: https://www.ncbi.nlm.nih.gov/pmc/articles/PMC3203180/

10. Nielsen EM, Björkman JT, Kiil K, Grant K, Dallman T, Painset A, et al. Closing gaps for performing a risk assessment on Listeria monocytogenes in ready-to-eat (RTE) foods: activity 3, the comparison of isolates from different compartments along the food chain, and from humans using whole genome sequencing (WGS) analysis. EFSA Support Publ. 2017;14(2):1151E.

11. Halbedel S, Prager R, Fuchs S, Trost E, Werner G, Flieger A. Whole-Genome Sequencing of Recent Listeria monocytogenes Isolates from Germany Reveals Population Structure and Disease Clusters. J Clin Microbiol. 2018;56(6).
